# Short Interspersed Nuclear Element (*SINE*) Sequences in the Genome of the Human Pathogenic Fungus *Aspergillus fumigatus* Af293

**DOI:** 10.1371/journal.pone.0163215

**Published:** 2016-10-13

**Authors:** Lakkhana Kanhayuwa, Robert H. A. Coutts

**Affiliations:** 1 Division of Biology, Faculty of Natural Sciences, Imperial College London, Sir Alexander Fleming Building, Imperial College Road, London, United Kingdom; 2 School of Life and Medical Sciences, Department of Biological and Environmental Sciences, University of Hertfordshire, Hatfield, United Kingdom; Woosuk University, REPUBLIC OF KOREA

## Abstract

Novel families of short interspersed nuclear element (*SINE*) sequences in the human pathogenic fungus *Aspergillus fumigatus*, clinical isolate Af293, were identified and categorised into tRNA-related and 5S rRNA-related *SINE*s. Eight predicted tRNA-related *SINE* families originating from different tRNAs, and nominated as *AfuSINE2* sequences, contained target site duplications of short direct repeat sequences (4–14 bp) flanking the elements, an extended tRNA-unrelated region and typical features of RNA polymerase III promoter sequences. The elements ranged in size from 140–493 bp and were present in low copy number in the genome and five out of eight were actively transcribed. One putative tRNA^Arg^-derived sequence, *AfuSINE2-1a* possessed a unique feature of repeated trinucleotide ACT residues at its 3’-terminus. This element was similar in sequence to the *I-4_AO* element found in *A*. *oryzae* and an *I-1_AF* long nuclear interspersed element-like sequence identified in *A*. *fumigatus* Af293. Families of 5S rRNA-related *SINE* sequences, nominated as *AfuSINE3*, were also identified and their 5'-5S rRNA-related regions show 50–65% and 60–75% similarity to respectively *A*. *fumigatus* 5S rRNAs and *SINE3-1_AO* found in *A*. *oryzae*. *A*. *fumigatus* Af293 contains five copies of *AfuSINE3* sequences ranging in size from 259–343 bp and two out of five *AfuSINE3* sequences were actively transcribed. Investigations on *AfuSINE* distribution in the fungal genome revealed that the elements are enriched in pericentromeric and subtelomeric regions and inserted within gene-rich regions. We also demonstrated that some, but not all, *AfuSINE* sequences are targeted by host RNA silencing mechanisms. Finally, we demonstrated that infection of the fungus with mycoviruses had no apparent effects on *SINE* activity.

## Introduction

Short interspersed nuclear element (*SINE*) sequences are short repetitive, non-coding sequences ranging in size from 100–600 bp. *SINE* sequences are widely distributed in eukaryotic genomes and have crucial roles in genome organization, genome evolution and modulating gene expression. *SINE* sequences have been implicated as being involved in cell survival during physiological stresses including heat shock, DNA damage, irradiation, oxidative stress, low temperature, exposure to toxic agents, and infection by pathogens or protoplast isolation [1–5]. *SINE* sequences are referred to as non-autonomous retrotransposons because they usually depend on enzymes encoded by long nuclear interspersed element (*LINE)* sequences for reverse transcription and retrotransposition. *SINE* sequences are transcribed by RNA polymerase III (pol III). Subsequently RNA pol III-*SINE* transcripts are reverse transcribed by reverse transcriptase (RT) and then re-integrated by endonuclease (EN) into various sites in the genome [6]. Some *SINE* sequences, which possess an intact RNA pol III promoter, are functionally active but the majority are not actively transcribed [6–8]. Large numbers of *SINE* sequences in eukaryotes are derived from tRNAs while others are derived from 5S rRNAs or 7SL RNAs [9].

To date *SINE* sequences have been reported in several fungi. For example *MgSINE*, isolated from the rice blast fungus *Magnaporthe grisea*, is a 472 bp tRNA-derived *SINE* present in *ca*. 100 copies in the genome which possesses features similar to mammalian *SINE* sequences [10]. Another *SINE* identified in *M*. *grisea* is the *mgsr1 SINE* which is an 800 bp element present in *ca*. 40 copies in the genome [11]. *Foxy* is an active *SINE* family found in *Fusarium oxysporum* f. sp. *lycopersici* strain Fo1007 and is present in *ca*. 160 copies in the genome [7]. *SINE* sequences have also been identified in several other filamentous fungi including *Egr1* (700 bp, *ca*. 50 copies; [12]) and *egh1 (EGH24*; 900 bp; [13]) both in *Erysiphe graminis*, *nrs1* (500 bp, 11 copies) in *Nectria haematococca* [14], *SINE2-1_BG* (a tRNA-derived *SINE*) in barley powdery mildew, *Blumeria graminis* [15, 16] and fifteen families of *infSINEs* sequences in the oomycete *Phytophthora infestans* [17].

*Aspergillus fumigatus* is a saprophytic and thermotolerant filamentous fungus which produces large numbers of asexual spores. However, the production of functional sexual spores (cleistothecia and ascospores) and its teleomorph *Neosartorya fumigata* have been described [18]. *Aspergillus fumigatus* is an opportunistic, airborne fungal pathogen that causes pulmonary invasive aspergillosis and is responsible for 90% of fungal infections in immunocompromised patients [19]. A non-LTR (long terminal repeat) retrotransposon *I-1_AF*, which belongs to the *Tad* clade of *LINE*-like element (LLEs), has been found in several copies in the *A*. *fumigatus* Af293 genome. These elements encode a DNA/RNA-binding protein, EN, RT and RNase H and insert randomly in the genome or precisely at the same target site in *Afut2_AF* [20, 21]. Recently a full description of the LLEs in several clinical and environmental isolates of *A*. *fumigatus* has been presented [22]. However, the existence of *SINE* sequences in the *A*. *fumigatus* genome is as yet not recorded.

The aim of this study was to investigate the occurrence of *SINE* sequences in the genome of the prototype *A*. *fumigatus* Af293 isolate. The abundance and distribution of *SINE* sequences were identified by interrogating the genomic DNA sequence for the occurrence of well-characterized signature motif sequences using computational analyses prior to mapping on the fungus genome and assessing potential transcription activity and silencing. Additionally the insertion patterns and copy numbers of the elements were compared in isogenic virus-free and virus-infected *A*. *fumigatus* isolates.

## Materials and Methods

### Computational Identification of *SINE* Sequences in the *A*. *fumigatus* Af293 Genome

#### Sequence dataset

The *A*. *fumigatus* Af293 reference genome sequence was accessed through the NCBI database and was also retrieved from several other online sources including the Central *Aspergillus* Resource (CADRE; [23]), the BROAD INSTITUTE *Aspergillus* comparative database and the *Aspergillus* Genome Database (*Asp*GD; [24]).

#### Search strategy and computational analyses

CENSOR software, developed by the Genetic Information Research Institute (GIRI; [25]) was used to interrogate the *A*. *fumigatus* Af293 genome sequences and screen for putative *SINE* sequences against a reference collection of repeats. Homologous portions of the query and reference sequences were masked and reports classifying all of the repeats that were discovered were generated. The RepeatMasker server (version: open-4.0) [26], which screens DNA sequences for interspersed repeats, was also used to screen the Af293 genome sequences against reference sequences in the Repbase libraries [27]. The reported sequences, which were classified as non-long terminal repeats (non-LTRs), *SINE* sequences, *SINE2*/tRNA and *SINE3*/5S were selected as *SINE* candidates and subjected to further analysis.

To identify tRNA-related *SINE* sequences, genomic sequences were examined using the tRNAScan-SE 1.21 program [28] to search for the presence of basic tRNA features such as RNA pol III promoter sequences and tRNA cloverleaf secondary structures using relaxed parameters and the EuFindtRNA algorithm. Subsequently selected sequences predicted from the program were resubmitted to tRNAScan-SE using the default settings. Sequences yielding positive reads were discarded as they were considered to be true tRNAs while the remaining sequences yielding negative reads and showed no support for a 5’-tRNA-related region, were considered for further analyses. The tRNA cloverleaf secondary structures and tRNA origin of the remaining sequences were also identified. In addition, the Genomic tRNA Database (GtRNAdb) was aligned with all the *A*. *fumigatus* tRNA sequences to separate tRNAs from candidate *SINE* sequences.

All predicted *SINE* sequences retrieved from Censor, RepeatMasker and tRNAScan-SE were submitted to BLAST analysis using accessible database sequences from the NCBI and *A*. *fumigatus* Genome Map Viewer to expand the boundaries of the masked sequences to 1,000 bp upstream and 2,000 bp downstream of both termini. Sequences with predicted tRNA-related structures were manually inspected for the presence of degenerate RNA pol III promoter A and B box sequences, extended 3’ tRNA-unrelated sequences upstream of an oligothymidine tract and also target site duplication (TSD) sequences flanking the *SINE* sequences. An additional check for the presence of the RNA pol III promoter sequence was performed by multiple sequence alignment of the predicted *SINE* sequences with the *A*. *fumigatus* Af293 tRNA gene sequences obtained from GtRNAdb. To identify 5S rRNA-related *SINE* sequences, sequences classified as *SINE3*/5S using Censor and RepeatMasker were aligned with the *A*. *fumigatus* 5S rRNAs and searched for the RNA pol III promoter (A, IE and C boxes) sequence. The process framework used for computational analysis of *AfuSINEs* is shown in S1 Fig.

Characterization, classification, distribution and location of *SINE* sequences was achieved by comparative analysis of the sequences. All predicted *AfuSINE* sequences were compared against the complete genome sequence of Af293 available on AspGD and CADRE by BLAST to search for locations of each element on the chromosomes. Multiple sequence alignments were performed using the Clustal Omega package [29] and MAFFT multiple sequence alignment software version 7 [30] and manual edition of the sequences. Phylogenetic trees were constructed using the neighbor-joining (NJ) method on MAFFT alignments [30].

### Fungal Strains and Culture Conditions

*A*. *fumigatus* Af293, which is a clinical strain, was used throughout the study. Af293 is naturally infected with a dsRNA mycovirus, Aspergillus fumigatus tetramycovirus-1 (AfuTmV-1), and NK125 is an isogenic, cured virus-free strain of the fungus [31]. A 20 μl suspension of fungal conidia was inoculated on Aspergillus Complete Media (ACM; [32]) agar plates and incubated for 4 days at 7C. Ampicillin (100 μg/ml) was added to the growth medium when required. For liquid culture cultivation, a 10 ml suspension of *ca*. 5x10^8^ conidia/ml was inoculated into 300 ml ACM broth and incubated at 37°C for 7 days on a rotary shaker at 150 rpm.

### Nucleic Acid Preparations

*A*. *fumigatus* genomic DNA was prepared using the DNeasy® Plant Mini Kit (QIAGEN, UK) using the mini protocol as described by the manufacturer. Total RNA extracts were prepared using the RNeasy® Plant Mini Kit (QIAGEN, UK) with the small scale protocol or Trizol as described by the manufacturers. DNA contaminants were removed from total RNA preparations using TURBO^TM^ DNase (Ambion, UK) prior to cDNA synthesis.

### PCR and RT-PCR Amplification of *A*. *fumigatus SINE* Sequences

Sequence specific primers were designed based on the sequences of candidate *SINE* sequences to cover the promoter regions, yielding amplicons *ca*. 100–150 bp in size (S1 Table). PCR amplification was performed under high stringency PCR conditions described as follows; 94°C, 5 min; 35 cycles of 94°C 30 sec, 55 or 60°C 30 sec and 72°C 30 sec; followed by final extension at 72°C for 5 min. RT-PCR amplification of *A*. *fumigatus* Af293 total RNA was performed using the same PCR cycling procedure and primer sets. Complementary DNA was synthesised using 10 μl of total RNA (*ca*. 50 ng/μl) using either random hexamers or sequence specific primers and Superscript III reverse transcriptase (Invitrogen, UK) following the manufacturer’s protocol. PCR amplicons were separated by gel electrophoresis and visualized by SYBR safe DNA staining.Southern Blot Hybridization of *A*. *fumigatus SINE* Sequence Amplicons.

Aliquots of 10 μg of genomic DNA were individually digested overnight with 1 μl of *Hind*III (New England Biolabs) at 37°C. Genomic DNA was also used as a DNA template for probe preparation and was labelled using the PCR DIG probe synthesis kit (Roche, UK) according to the manufacturer’s instructions. Restriction fragments of genomic DNA were separated by electrophoresis on 1% w/v agarose gels containing SYBR safe DNA stain in 1XTAE. Gels were denatured in 0.25 N HCl and 0.5M NaOH + 1.5 M NaCl, followed by neutralization in 0.5 M Tris-HCl + 1.5 M NaCl. The DNA was then transferred onto a positively charged Amersham Hybond^TM^-N membrane (GE healthcare) and nucleic acids were subsequently fixed by UV-cross linking prior to probing.

### Detection of Small RNA Molecules Homologous to *A*. *fumigatus SINE* Sequences

**Fig 1:**
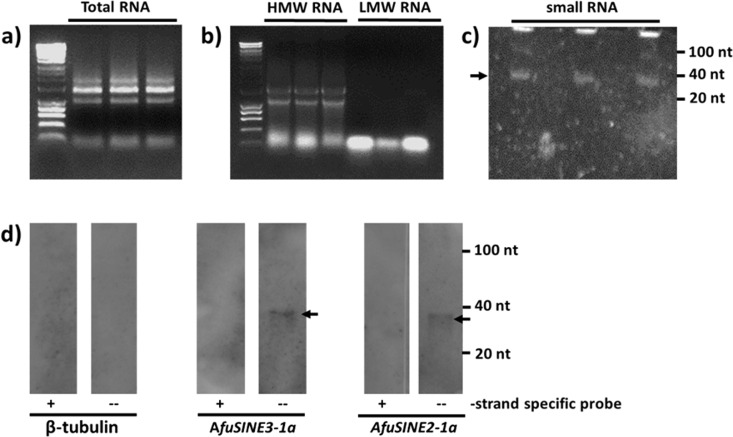
Detection of small RNA molecules homologous to *AfuSINE* sequences. Total RNAs isolated with Trizol were electrophoresed on 1.5% agarose gels (a). After separation by PEG precipitation, high molecular weight RNAs (HMW RNAs) and low molecular weight RNAs (LMW RNAs) were electrophoresed on 1.5% agarose gels (b). The LMW fractions from 400 ng/μl of total RNA were resolved on 15% (w/v) polyacrylamide Tris-borate-EDTA-urea gels and stained with SYBR Gold Nucleic Acid Gel Stain (c). Northern blot hybridization of small RNAs homologous to *AfuSINE* sequences in *A*. *fumigatus* Af293 (d). LMW RNA fractions were isolated using TRIzol and 5 μg RNA sample was loaded into each well for northern blot analysis. Only small RNAs (<40 nt; arrowed) from the antisense strand were detected.

Small, low molecular weight (LMW) RNAs were isolated using the procedure of Lu et al. [33] with some minor modifications. Total RNA, high molecular weight RNA (HMW RNA) and LMW RNA fractions were analyzed on 1.5% agarose and 15% (w/v) polyacrylamide Tris-borate-EDTA-urea gels (Bio-Rad, Sweden) (Fig 1A–1C). For the detection of homologous *SINE* small RNAs, 100 μg of the small RNA fraction was separated on polyacrylamide gels. For northern blot hybridization of small RNAs, nucleic acids were transferred to nylon membranes and fixed using 1-ethyl-3-(3-dimethylaminopropryl carbodiimide; EDC)-cross linking [34]. Prior to hybridization, probes were cleaved to an average length of 50 bp by alkaline hydrolysis as described by Kreuze *et al*. [35].

### Secondary Structure Prediction of *A*. *fumigatus SINE* Transcripts

The secondary structures of *SINE*s were predicted using Mfold [36] using default settings with a folding temperature set to 37°C, which is the optimal growth temperature of *A*. *fumigatus* Af293. RNA structures showing the lowest free energies were accepted as the most likely structures for the *AfuSINE* sequences.

## Results and Discussion

### Search Strategy and Identification of *AfuSINE* Sequences by Computational Analyses

Here we report for the first time the presence and distribution of *SINE* sequences in the *A*. *fumigatus* Af293 genome by exploiting the availability of its complete genome sequence to investigate their incidence. In this study, a computational search strategy was developed to predict the occurrence of *A*. *fumigatus SINE* sequences (designated as *AfuSINE* sequences) using several bioinformatic software packages. The Af293 genomic sequences were initially screened for the interspersed repeat consensus of *SINE* sequences using Censor and RepeatMasker against the available sequences from the reference collection of repeats in the Repbase database. The tRNAscan-SE search server was also used to screen for tRNA-related *SINEs* using relaxed parameters. In total 17,269 candidate sequences were retrieved. These include simple repeats, small RNAs, tRNAs, 5S rRNAs, 7SL RNAs, tRNA- and 5S rRNA-related sequences, sequences homologous to *SINE* and *LINE* sequences as well as other transposons. However, since these programs identify exact sequence matches, any data retrieved required further screening with the tRNAscan-SE program using default parameters to eliminate true tRNA sequences. The 5S rRNA sequences were eliminated by BLAST analysis against Af293 5S rRNAs on the NCBI database. Subsequently, the sequences that do not exhibit *SINE* characteristics were discarded, leaving 145 candidate sequences nominated *SINE2* sequences (tRNA-derived *SINE* sequences) and *SINE3* sequences (5S rRNA-derived *SINE* sequences) for further investigations including the extension of *SINE* boundaries, BLAST analysis, and inspection of 5S rRNA-related *SINE* and tRNA-related *SINE* features.

Following this analysis, thirteen candidate *SINE* sequences were identified which were divided into *AfuSINE2* and *AfuSINE3* types. Most of these sequences possessed features in common with generic *SINE* sequences but some lacked TSD and intact internal RNA pol III sequences. However some did contain masked sequences similar to *SINE* sequences from a broad spectrum of reference organisms including *A*. *oryzae*, *A*. *nidulans*, *B*. *graminis*, *Cryptococcus neoformans*, arabidopsis, hedgehogs, mosquitoes and humans. The organization and structure of representative *AfuSINE* sequences are shown in Table 1 and S2–S5 Figs.

**Table 1:** List of 5 putative 5S rRNA-related *SINE* sequences (*AfuSINE3*) and 8 putative tRNA-related *SINE* sequences (*AfuSINE2)*.

Chr.#	AfuSINEs	AfuSINE sequences	Origin	Length (bp)	Activity	Estimated copy no.
1	**AfuSINE3-1a**	TAGGCTCCAACCAAGCACATACGACCATAGGGTGTGGAAAACAGGGCTTCCCGTCCGCTCAACCGT**ACTTAAGCCAGGTG**TAGAAT**GTGGT**CAATCCA**TAGAAAGCCCTTTTGCCT**CCAAAGCAGCGGCGGGATGCTTTTCGTATGAGTGCTGGTGATGTGGGTGAGAACTCCAGGGGGCCCCATCACCCCTGGTGATGATGGCTCTAGGGGTTTGATGAAGGCTAACTTAACAGCTGACTGCAGAAGAAAACAGTGATTCAGCTGCTTTTAATAGGCTC	5S rRNA	280	Active	5
3	**AfuSINE3-3a**	AGCTTGAAAACAAGCACATACGACCATAGGGTGTGGAAAACAGGGCTTCCCGTCCGCTCAGCCGT**ACTTAAGCCACATG**TATCAAC**GTGGT**CGGAAGT**TATATGCGCCCTTAATGG**CCTGCTGCTTTACGGGTGAGAGACGCTAAACCATAGGGTGAGTGTGGGTGAGTGAAGGTGAGCTGAGTGATGCTATCCAACGCCATGGGTGAGCAAGGGTGATCAGTGGGTGACAGTGTGATTCTATAATGACTAGACTCCGTCTATCGCTAGCTTAAA	5S rRNA	275	Inactive	NA
3	**AfuSINE3-3c**	ATTCAGTTATCTTTCTCCCAGCACATACGACCATAGGGTGTGGAAAACAGGGCTTCCCGTCCGCTCAGCCGT**ACTTAAGCCACACG**TAGAAC**GTGGT**CAATCCC**TAGAAAGCCTTTTTGCTT**CTAAAGCAGCGGCGTAATTCTTTTAGTATGAGTGCTGGTGATACGGGTGAGGACCCCGTCACCCCTGGTGATGATGGCTTACTGGTGGTGACGAGGGCTCCAGGGGGGTGACGAAGGCGTACTCAGAAGTCAACCACAGAAGATACAGTGATTGAGCTACTTTTAATGGGCTCTGCGCCCCGCAGAAGTAATGCACTTGATTATTCCTGAGTCCATACAGT	5S rRNA	343	Active	5
4	**AfuSINE3-4a**	CGTCAGATTGCACAATGACCATAGGGTGTGGAAAACAGGGCTTCCTGTCCGCTCAGCCGT**ATTCAAACCAGTAT**TATTAAT**TTGGT**CGGAAGT**TATATGCGTCCCTAAAGG**CCTGCCGCTCTACGGATGAGAGACACTGAACCATAGGGTGAGTGTGGTGAGTGAAGGTGAGCAGAACGATGCTATCTATTGAATTATTCACATCGGCGATCCGTAGAACAGGTTGGTCCCACGTCCTGGACACGCCCATACTCCGTCA	5S rRNA	259	Inactive	NA
5	**AfuSINE3-5c**	AGCTTCTATATGAATTTCTTGCACGAATATCCTAGCACATACGACCATAGGGTGTGGAAAACAGGGCCTCCTGGCTGCTCGGCCAT**ACTTAAGCCAGTAT**TATTAAT**ATAGT**CAAAAGT**TATATGCACCCTTAAAGG**CCTGCTACTTTACAGATAAGAGATACTAAACCATAGGGTGAGTATAGGTGAGTAAAGGTGAGCTAAGTAATGCTATCTAACAGCATACTTGAGCATGGTGATTAGTAGATAACAGAGTGATTCTATCATGACTAGACTCCTCTATCACTAGCTTCAATAT	5S rRNA	297	Inactive	NA
1	**AfuSINE2-1a**	AAGTGTACATAGAGCCGGTAGCGG**TAGCGTAGTGG**TAAGCGCTCCGAGGCAGCCTCTAGAGAAGTAG**GATTGGTGACC**CTGCTTTTTTGGAGGTTATG**GGTTCGATTCC**CGTCGCTGGCACA**ACATT**TACCACCACAATGGAAGATCACTTCCCACAATGGTATCAAGGCCACTCCCTTATCGCAAGGTGGTGGGGAAGTTGGAACAATCACAGGCCTGTAAGGCGAGGCTCTAAATTCGCCCTCATGTAATGGAACAAAATGTAACTAGACACACAAGGATTAGCTATAGTCGATACCTGCATATCGCCCAAGGCGAGGGGTCAGCGTATGAGT***ACTACTACTACTACTACTACT***AAGTGTACATAGAG^A^	tRNA/Arg	370	Active	4
3	**AfuSINE2-3a**	AGGGAGTTTGATATCCCATTT**TGGAGGGACTGG**CCGGCTCTGGGGTCGGTCGTTAAGGCGCTCCGCCATTCATCTGCA**GGTTGGATAAC**AGCCGGGCAATTTTCGTGACTGAAAATCCCATCCATCTGAAACTCTTGTGAGTATGTTGAAAGCCGACTTTTCAAGACGCCAATCTCAGGGCCATCCGCAGCTGAAAGATAACTCTCGAAGCAATGCCGGCACTGTAGAAAGCAGCAACGGAGAAGAGGCGATTGTTGGTGTCCGGCCAACTTGACATAACGATCGAATAGATCGA**ACATT**CGTTGCGAGGTTTCGACGGCCAGATCAATCATATCGGCACTGGGAGACCGACAGTGGCGGACAAGTGCGAGAATCACAAAGACGGCTGTTAGATGCAAGGTAAGAAGAGGCGGCGTAGTAGAGTCGGACCTGGGATTTGAGTATTGTGCTAGAAGAATCGCCTGTTGTTCCTGGATCTGGAGCAGGAGGGAGT^B^	tRNA/Ser	493	Active	1
4	**AfuSINE2-4a**	ATGTACATCAGGTGTA**TGCGGCCTGG**GCAGAAATATGATGGGTAGATATCAAGATATC**GTAAGTATTCC**AGCTCCT**CCA**CTGATTGACGATCCTTTTGTTATCTTGAGCCAAGGAATTTGGTGCCTTTGGGCCTGAGGTTCCAGATCCTCCCCCACATCCGCGAACCTCATTTCGTGGTTAAGGACA**ACATT**AAATAACCGTGAATTTACTCCGTAGTGTCTCGACCCTTTGGGGGGTGATCTTGTAAAGATTAGGAAAGAATGAGAGAAATAAATCACAGATTTTTTTTTTTTTTTTTTCGTAAGAAAGAAAAGGCAACTCCCTCGTCACAGCAGGATTTATGTACAT^C^	tRNA/Ser	349	Active	1
7	**AfuSINE2-7a**	TCAAAACGCTGGAACTGGGTTTATTTCCATT**TGGCGGAATGG**AAAGGTTCGAAACAGCCCTGGAGG**TGTTCGAATAG**TAGAGTATGGTCAGCTTCGCTTCCTGCTCGTGTGACTATGATGTAAAGATGTTGGGTTATTTTATTTCAATCAATCAGTGGGCCGCATTCTATTCTTACAAGTCCCTTCAATGGCAATCATGGATATCAGATGGAGAGATTTGCGATGCTTCTGTGTCTCTAATAATAAGCAGTCTTCAGATGAGTGGAGGCGGCAGAGAAGGGGAGCAATGGGGACCAATGGGGAGCAATGGGGAGTAATAAGGAGCAACAAGGATGAAGAGGATTGGTGTGACGACCCCTGCATACAACACTTGTAGGAAACGACATCTTATCAATCTCAAA	tRNA/Gly	401	Inactive	NA
5	**AfuSINE2-5d**	TTTTTAGTTGCTACGT**AGGCTTAATGG**AAGGAACATGTTCGAGGATGGTCGGAAAATCGCTGGAGG**TGTTCAATCCC**CGAGGTACGTCATCTGGAACTGAATCAAACCTCT**CCA**CTCGAGGGAGAGCTGAGAGTATTAGGTGGTTTTCGTACTTATATCCAAGGCTCATTATCACGAATGCCGTACAAACAAACACAATCGAGATACTCCATACTTGAATTATTTTCAGT	tRNA/Ser	230	Active	2
3	**AfuSINE2-3c**	TGTGATGCCTTTCCACACTGCATGGTTGTCTCGGCACAGA**TGGCAAAGCCGT**TCCTGGCAACAGTTAATTGTTC**AGTTCAAATCT**GTTAAAACGGGGTCGTACTTACCATAGACTGTCGTCGAGCATCTGCCGCAGCTCTAGCCAAAAGACCCAGTGATTTCTGCCTATGAATGACAGGTCAGCTTTGTGGGGGCTTTGCA	tRNA/Ala	201	Active	5
4	**AfuSINE2-4c**	CTTGGAGATTACATACGTA**TCCTGCAATGG**GCAGAATCAACAACGTGTACATTTTACGCGTAAAT**GGATCGATTCC**ATATGTA**CCA**CTCTCTAAGCCAACGCAACCATGAAAGGGTGAGAAGACCCTGACGGCCACCGAAACACTTACCAACCAGTTCTTGGTTGTGAACATAGTATGGATAATTTGGTAGCTATAGTTGATTTGAATAATGGTGCTGTCGCACCTCGCAATGTCTTTTTAGCATCTTGGAGA	tRNA/Thr	253	Inactive	NA
7	**AfuSINE2-7e**	AATTACTTATA**TAGCAGAGTGG**TAGAGTCATAGGCTTCATATTTTTACAGCAATGGAGATGAGATAAG**GATTCGATTTC**TATATATTTTAGTACTAGATAATATTAACTTTAAGGGAAGCTATAGGATTTTCCCCTAATT	tRNA/Met	140	Inactive	NA

**Annotations:** **NNNN** = RNA pol III promoter sequences; NNNN = target site duplication (TSD);***NNNN*** = short repetitive sequence

**NNNN** = 5' tRNA-related CCA end; **NNNN** = ACATT at 3’ end

^A^ Sequence underlined by dotted line is similar to *I-4_AO*#*LINE*/Tad1 of *A*. *oryzae* (Galagan et. al., 2005; Kapitonov and Jurka, 2006) and *I-1_AF A*. *fumigatus* Af293 *LINE*.

^B^ The 5’ tRNA-related region is similar to a 481 bp *LFSINE_Vert* (*SINE2*/tRNA#His) identified in *Latimeria menadoensis* (Bejerano et al., 2006).

^C^ The 5’ tRNA-related region is similar to a 268 bp *MIRc* (*SINE2*/tRNA#Pro) identified in mammals (Smit and Riggs, 1995).

#### 5S rRNA-related *SINE* sequences (*AfuSINE3*)

Families of five 5S rRNA-derived *SINE*-like sequences (*AfuSINE3*) were discovered which are homologous to the 5’-terminal region of fungal 5S rRNA sequences. The elements are present in five related copies following *in silico* analysis (nominated *AfuSINE3-1a*, *AfuSINE3-3a*, *AfuSINE3-3c*, *AfuSINE3-4a* and *AfuSINE3-5c* on chromosomes 1, 3, 4 and 5, respectively) with an average length of 259–343 bp. All five *AfuSINE3* sequences are related to *SINE3-1_OA*, which is a 5S rRNA-derived *A*. *oryzae SINE* (Table 1 and S4 Fig).

Pairwise alignment of the *AfuSINE3* sequences with 5S rRNAs revealed that the 5’-terminal head regions (positions 1 to 64) are well-preserved and are very similar to the sequences of *A*. *fumigatus* 5S rRNA (S4 Fig). Significant similarity was also observed when the 5’-terminal head together with portions of the body and tail of the *AfuSINE3* sequences were aligned with *A*. *oryzae SINE3-1_AO* (S4 Fig) which in turn is 50–65% homologous and 60–75%% similar to 5S rRNA gene sequences. With at least 60% similarity to the 5S rRNA species and a 60-nucleotide overlap, these observations suggests that all *AfuSINE3* sequences are indeed derived from 5S rRNA.

**Fig 2:**
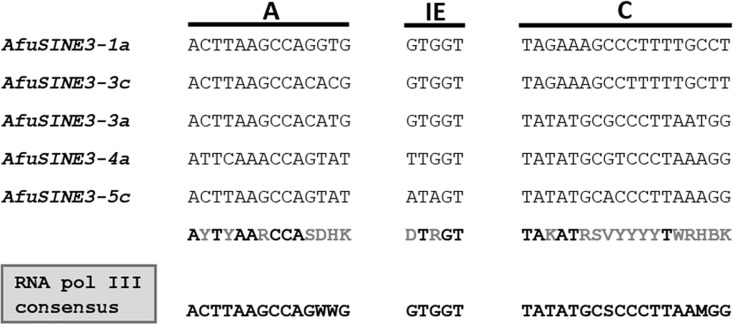
Consensus sequences of A, IE and C box promoters of type I RNA pol III in 5S rRNA-derived *AfuSINE* sequences (*AfuSINE3s*).

A comparison of the five *AfuSINE3* sequences (positions 1 to 119) revealed similar conservation at the 5’-terminal head sequences when some parts of the body regions were included (S6 Fig). However excluding the upstream sequence resulted in significant similarity only being found at the beginning (24 nt) of the 3’-terminal divergent body sequence of the five *AfuSINE3* candidates (S7 Fig). Regions of three type I RNA pol III domains (A, IE and C boxes) were poorly conserved in *AfuSINE3* as compared to typical 5S rRNAs (Fig 2 and S8 Fig). Additionally, the *AfuSINE3* sequences lack a termination signal for RNA pol III transcription (GCTTTTCG) apart from *AfuSINE3-1a*. This feature may result in continuous transcription activity extending the 5S rRNA-related region towards the 3’ termini of the *AfuSINE3* sequences.

Phylogenetic analysis showed that *AfuSINE3-1a* and *AfuSINE3-3c* cluster in the same clade as *SINE3-1_OA* (S9 Fig). Two main considerations used to define an *AfuSINE* family in this study are 1) their same common origin and 2) their same sequence module/structure. However, the terminal (tail) sequences of some familial *SINE* sequences were variable and thus these were omitted from the *AfuSINE* phylogram. From these criteria and the alignment of the *AfuSINE3* candidate sequences were considered to have a common origin since the alignment showed similarities in the 5’-terminal head and some parts of the body regions. Phylogenetic analysis showed similar results, revealing all five predicted *AfuSINE3* sequences grouped into two closely related, distinct lineages. Thus, it can be inferred that *AfuSINE3* sequences originate from the same gene.

#### tRNA-related *SINE* sequences (*AfuSINE2*)

Eight tRNA-derived *SINE*-like sequences (*AfuSINE2*) originating from different tRNAs were identified which ranged in size from 140–493 bp. In order to identify internal RNA pol III promoter sequences (A and B boxes) with a 10–11 bp consensus sequence, the 5’-terminal regions of the *AfuSINE2* sequences were aligned with *A*. *fumigatus* Af293 tRNA sequences. However, both the *AfuSINE2* RNA pol III A and B box promoter sequences were degenerate with the former more degenerate than the latter.

**Fig 3:**
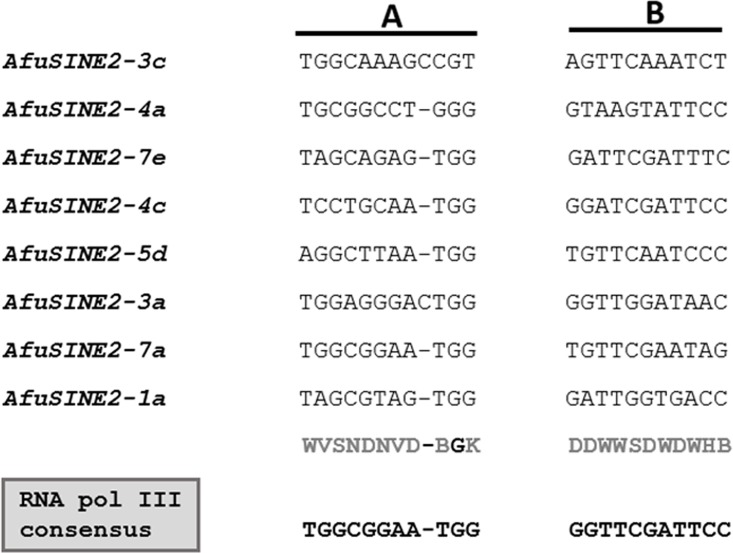
Consensus A and B box promoter sequences of RNA pol III in tRNA-derived *AfuSINE* sequences (*AfuSINE2s*).

Consensus sequences of the RNA pol III promoter A and B boxes of tRNA-derived *AfuSINE* sequences are shown in Fig 3. The 4–14 bp long TSD repeat sequences (a characteristic feature of SINE genomic insertions) were partially degraded in some *AfuSINE2* sequences with 1 or 2 residues missing e.g. in *AfuSINE2-3c* and *AfuSINE2-5d*. This degradation phenomenon might reflect the age of the *SINE* and concern nucleotide substitution over many generations, over an extended period of time [17]. As a consequence it’s possible that *AfuSINE2-1a* might have emerged more recently that the other *AfuSINE2* sequences since it possessed intact direct repeats. It has been documented that *SINE* insertion into the genome usually produces short 5–8 bp TSDs at the 5’- and 3’-termini of the sequence. However if *SINE* insertion is very ancient, TSD identification might be difficult due to mutations in the sequence. Also a TC motif located immediately upstream of the A/T-rich tail and an additional B box (B’ box) downstream of the RNA pol III promoter were absent from most of the *AfuSINE2* sequences except *AfuSINE2-1a* which contained an additional B’ box GGTTCGATTCC sequences 20 bp downstream of the B box. Interestingly this feature has been reported in some tRNA-related *VES SINEs*, *P*.*k*. *SINE*s in bats and SINE B1 in rodents [37, 38, 39].

Additionally, none of the 5’-terminal regions of the *AfuSINE2* sequences could be folded into tRNA-like cloverleaf structures, which is in contrast to the *MgSINE* and the *MIR SINE* sequences [10, 40].

No significant similarity was observed among the predicted *AfuSINE2* sequences and they appear to originate from different tRNA-related sequences (S10 Fig). The ancestral tRNA of each *AfuSINE2* was identified using the tRNAscan-SE and multiple sequence alignment of the predicted tRNA sequences. The results indicated that the five *AfuSINE2* sequences are similar in sequence to the 5’-tRNA-related regions of respectively tRNA^Ala^, tRNA^Arg^, tRNA^Gly^, tRNA^Met^, tRNA^Thr^and tRNA^Ser^. Phylogenetic analysis revealed that the predicted *AfuSINE2* sequences do not group into distinct lineages as the bootstrap supports are too low to statistically support any of the nodes (S11 Fig).

### Similarity of *AfuSINE* Sequences to Retrotransposons

**Fig 4:**
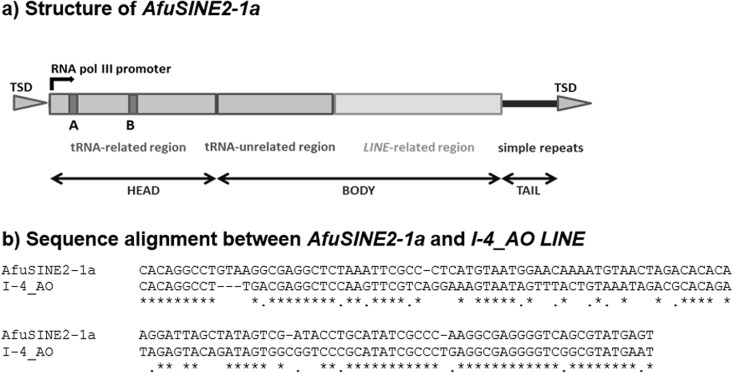
Structure of the *AfuSINE2-1a* tRNA-derived *SINE*. Structure of the *AfuSINE2-1a* tRNA-derived *SINE* found in *A*. *fumigatus* Af293 (a) and the alignment of its 3'-terminus which is related to *I-4_AO LINE* (b).

Interestingly the *AfuSINE2-1a* 3’-terminal sequence was similar to that of *LINE*, *I-4_AO*#*LINE*/*Tad1*, identified in *A*. *oryzae* [20, 21] following CENSOR analysis (Fig 4). Subsequently, the element was aligned manually with retrotransposons found in the *A*. *fumigatus* Af293 genome such as a retrotransposon-like element (*Afut1-LTR*; [41]) and a non-LTR retrotransposon (*I-1_AF*; [20, 21]). The analysis revealed that the 3’-terminus of the *AfuSINE2-1a* at positions 210–335 appears similar to the 3’-untranslated region (UTR) of the *I-1_AF LINE*-like sequence which terminates the RT gene in ORF2. It has been proposed that sequence and structural similarity of the 3’-terminal region of tRNA-derived *SINE* sequence with a corresponding *LINE* sequence is crucial for its retrotransposition [42]. Since *AfuSINEs* are non-autonomous retrotransposons with no gene coding capability, the elements may need to exploit the action of other retrotransposons (such as *LINEs*) for their amplification and insertion into the genome. Thus, the presence and transcription activity of retrotransposons such as *I-1_AF LINE* sequences could signify the occurrence and activity of *AfuSINE2-1a*. Additionally, it was noted that the 3’-terminus of *AfuSINE2-1a* terminates with a series of short repetitive ACT trinucleotide sequences.

### Distribution and Location of *AfuSINE* Sequences

**Fig 5:**
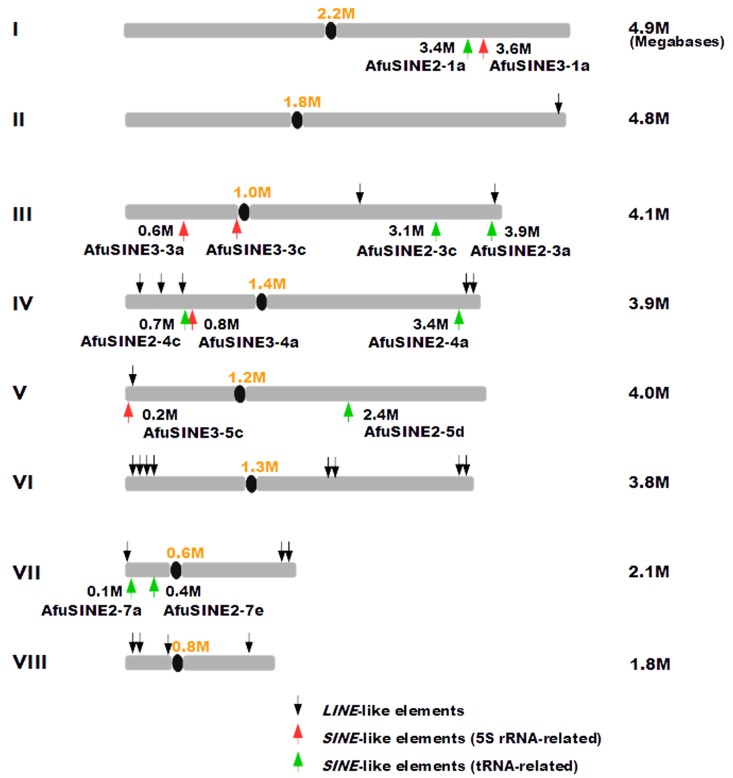
Mapping of *SINE*-like sequences of the *A*. *fumigatus* Af293 genome. Mapping of *SINE*-like sequences on eight chromosomes of the *A*. *fumigatus* Af293 genome including previously described *LINE*-like sequences (LLEs; [22]). Chromosome numbers are shown on the left and the size of each chromosome is shown on the right.

The distribution and location of *AfuSINE* sequences on the eight chromosomes of the *A*. *fumigatus* Af293 genome were investigated by a comparative analysis of the sequences. Computational analyses revealed that *AfuSINE* sequences are not abundant in the fungal genome (Fig 5). *AfuSINE* sequences are dispersed on chromosomes 1, 3, 4, 5 and 7, and are more abundant on chromosomes 3 and 4. The elements are randomly dispersed in pericentromeric and subtelomeric regions on the chromosomes and inserted within gene-rich regions, normally in intergenic regions or close to coding regions similar to LLEs [22]. These observations suggest that the insertion of *SINE* sequences and *LLE* sequences in *A*. *fumigatus* chromosomes may be linked.

**Fig 6:**
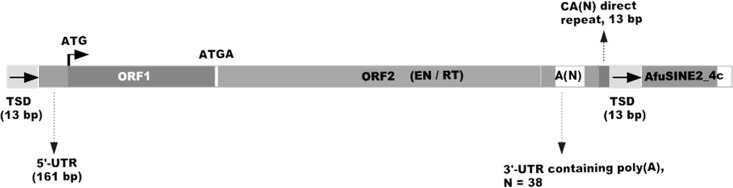
Structure of *LINE*-like element. Structure of *LINE*-like element (LLE#4_3.0; [22]) on *A*. *fumigatus* Af293 chromosome 4 showing insertion of the *AfuSINE2_4c* sequence next to the LLE RT.

Interestingly, *AfuSINE2_4c* on chromosome 4 appears to be located very close to a *LINE*-like retrotransposable sequence (LLE#4_3.0); a subclass previously identified in the *A*. *fumigatus* Af293 genome [22]. The LLE#4_3.0 is a non-LTR retrotransposon from *I* clade which is flanked by 13 bp TSDs and contains two overlapping ORFs (open reading frames) ORF1 and ORF2. ORF1 encodes a 413-aa DNA/RNA-binding protein (pos. 175–1669) and ORF2 encodes a 1273-aa polyprotein (pos. 1666–5487) encoding an EN and a RT. The insertion of *AfuSINE2_4c* next to the RT of LLE#4_3.0 *LINE*-like element (Fig 6) suggests that activity of *AfuSINE2_4c* possibly relies on this *LINE*. However, *AfuSINE2_4c* is transcriptionally inactive possibly because of inactivation of the LLE#4_3.0 RT domain by mutation. Since most of the LLE sequences identified in the genome of *A*. *fumigatus* Af293 are not intact, this could potentially contribute to loss of transcription and retrotransposition activities for some *AfuSINE* sequences.

### Transcription Activity of *AfuSINE* Sequences in the Genome by RT-PCR

Initially PCR amplification was performed using genomic DNA as a template under high stringency PCR conditions. The results of these experiments showed the expected amplicons for the thirteen candidate *AfuSINE* sequences (S12 Fig, lane 1 throughout) proving the existence of the sequences on the *A*. *fumigatus* Af293 genome and confirming that the genomic information in the database is correct. Additionally, a ladder of DNA amplicons of increasing size was not found following PCR amplification indicating that *AfuSINE* sequences are not present as an array on the genome.

Subsequently, RT-PCR amplification was performed using total RNA with the same sets of primers to investigate the production of *AfuSINE* transcripts. The results illustrated that seven out of thirteen *AfuSINEs* are transcriptionally active (*viz*. *AfuSINE3-1a*, *AfuSINE3-3c*, *AfuSINE2-1a*, *AfuSINE2-3a*, *AfuSINE2-3c*, *AfuSINE2-4a* and *AfuSINE2-5d;* S12 Fig, lane 2 throughout). No amplicons were observed from the cDNA (-RT) samples, indicating that contaminating genomic DNA was absent from the reaction mixtures for cDNA synthesis (S12 Fig, lane 3 throughout). All RT-PCR amplicons were sequenced to confirm their identity. These results illustrated that the levels of transcription of the *AfuSINEs* are very low as compared to the β-tubulin gene which is a constitutively expressed house-keeping gene. Additionally RT-PCR analysis clearly demonstrated that not all of the *AfuSINE* sequences in the *A*. *fumigatus* Af293 genome are transcribed.

### Estimation of *AfuSINE* Copy Number in *the A*. *fumigat*us Af293 Genome

**Fig 7:**
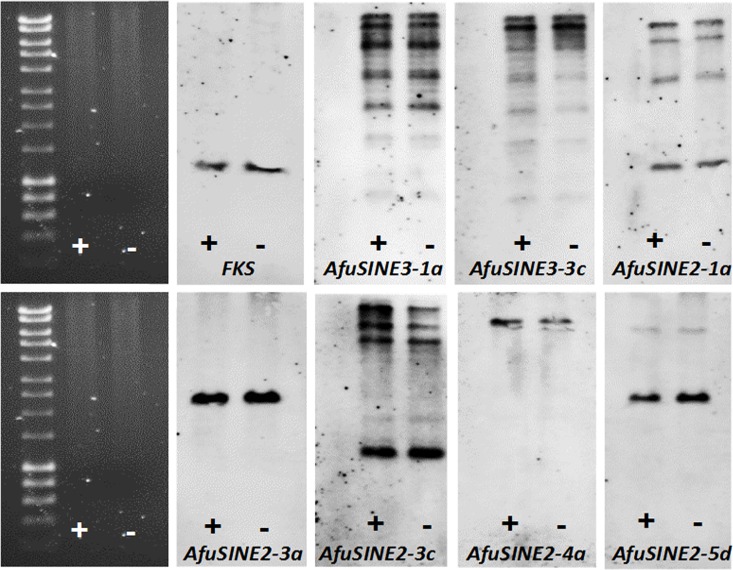
Southern blot hybridization of *AfuSINE* sequences in the genome of *A*. *fumigatus* Af293. Southern blot hybridization of *AfuSINE* sequences in the genome of *A*. *fumigatus* Af293 (virus-infected strain; indicated by plus sign) and *A*. *fumigatus* NK125 (virus-free strain; indicated by minus sign). *Hind*III-digested DNA of each strain was separated in 1% agarose gels in 1xTAE, denatured and blotted onto nylon membrane. Hyperladder 1 (M; 10 kbp; Bioline) was used as marker.

Only actively transcribed *SINE* sequences *viz*. *AfuSINE3-1a*, *AfuSINE3-3c*, *AfuSINE2-1a*, *AfuSINE2-3a*, *AfuSINE2-3c*, *AfuSINE2-4a* and *AfuSINE2-5d* were investigated in this study. The copy numbers and transposition of the seven *AfuSINE* sequences above per genome of two isogenic *A*. *fumigatus* Af293 lines, one infected with AfuTmV-1 and one virus-free (NK125; [31]) were examined by Southern blot hybridization. *A*. *fumigatus* Af293 genomic DNA was digested with *Hind*III which does not restrict the *AfuSINE* sequences, thus it can be assumed that any single hybridizing band observed corresponds to one copy of each *AfuSINE*. Southern analysis of the *AfuSINE2* sequences showed strong, single hybridization signals for the *AfuSINE2-3a*, *AfuSINE2-4a* and *AfuSINE2-5d* sequences indicating that these elements are present as single copies in the genome (Fig 7). Four or more strong hybridization signals were detected with *AfuSINE2-1a* and *AfuSINE2-3c*, indicating that at least four copies of these elements are present in the genome. At least five strong hybridization signals were detected for both *AfuSINE3-1a* and *AfuSINE3-3c*, indicating several copies of each *AfuSINE3* sequence dispersed in the genome. The *A*. *fumigatus fks* gene, which is present as a single copy, was used as a control in these experiments.

These results indicate that *SINE* sequences in *A*. *fumigatus* are present in very low copy number. For instance *AfuSINE3* sequences are present as *ca*. five copies which is in contrast to other eukaryotic *SINE* families where *ca*. 10^4^ copies of *SINE3* types are present in the zebra fish genome [8] and in *M*. *grisea* the *Mg-SINE* where the copy number was estimated to be *ca*. 100 [10]. Because of the known and significant divergence in the Af293 *SINE* sequences and potential insertions and/or deletions within them it is likely that the copy numbers estimated here are underestimates and represent the minimum copy numbers for each element. It is also likely that *A*. *fumigatus SINE* RNA transcripts are silenced since Aspergilli encode all of the enzymes required for the RNA silencing process and are active *in vivo* [43]. As the number of repetitive elements in *A*. *fumigatus* are limited, this could contribute to the small size of the *A*. *fumigatus* genome (29.4 Megabases) as compared to for instance the oomycete *P*. *infestans* genome which consists of a large proportion of TEs and repeats contributing to a genome size of 240 Megabases [17]. Thus, *SINE* abundance is clearly diverse and variable between different fungal and oomycete species. Low copy number non-LTR retrotransposons with degenerate sequences are likely to be lost from the genome as a result of genetic drift and natural selection [44].

Infection of *A*. *fumigatus* with mycoviruses causes some effects on retrotransposon activity. For instance in *A*. *fumigatus* strain A56, it has been demonstrated recently that chrysovirus infection stimulates LLE mobilization [22]. However infection of *A*. *fumigatus* strain Af293 with AfuTmV-1 had no obvious effects on *AfuSINE* transposition or copy number since both virus-free and virus-infected isogenic lines showed identical hybridization patterns (Fig 7).

### Detection of Small RNA Molecules Homologous to *AfuSINE* Sequences

To confirm *AfuSINE* regulation and gene silencing in *A*. *fumigatus* Af293, small RNAs homologous to *AfuSINE* sequences were identified following northern blot hybridization. DIG-labeled probes for *AfuSINE* sequences were *in vitro* transcribed in both sense and antisense orientation to detect strand-specific small RNAs. In this study, only four representative actively transcribed *AfuSINEs* (A*fuSINE3-1a*, *AfuSINE2-1a*, *AfuSINE2-3a* and *AfuSINE2-4a*) were selected for detection of small RNAs.

Autoradiographic analysis showed weak hybridization signals of a band corresponding to <40 nt for the antisense (-) strands of A*fuSINE3-1a* and *AfuSINE2-1a* (Fig 1D) while small RNAs homologous to *AfuSINE2-3a* and *AfuSINE2-4a* were not detected. Constitutively expressed control β-tubulin probes were also used to detect small RNAs homologous to the β-tubulin gene. No hybridization signals were found in the controls, suggesting that hybridization of small RNAs with *AfuSINE* probes was not attributable to mRNA degradation.

The presence of small RNAs (<40 nt) homologous to *AfuSINE3-1a* and *AfuSINE2-1a* sequences suggests that these elements may be targeted for degradation and silencing in the fungus. However, small RNAs were not detected with *AfuSINE2-3a* and *AfuSINE2-4a* suggesting that not all *AfuSINEs* are targeted by host RNA silencing. In addition, hybridization signals observed from the northern blot analysis are very weak possibly attributable to low transcription activity of the elements resulting in low abundance of siRNAs.

In summary, our study has demonstrated the first computational search for *SINE* sequences in the *A*. *fumigatus* Af293 genomic DNA. Distribution, copy number, transcription activity and silencing of these elements have been described. Further research is required to investigate *SINE* distribution among other clinical and environmental *A*. *fumigatus* isolates and to assess the effects of *AfuSINE* sequences on adaptability and pathogenicity. Potentially active *AfuSINE* sequences, for example *AfuSINE2-1a*, might be further developed as a reverse genetic tool. The fact that this element was actively transcribed, possessed intact 14 bp TSDs, a 5’-tRNA-related region corresponding to tRNA^Arg^ as reported for most active tRNA-derived *SINE* [38, 45, 46], sequence similarity to the *I-1_AF LINE*-like element and was a target for host silencing illustrates its potential.

## Supporting Information

S1 Fig Processing framework used for computational analysis of *AfuSINEs*. (TIF)

S2 Fig Structure of the representative *AfuSINE3* sequences. Predicted secondary structure was performed using Mfold program (http://mfold.rna.albany.edu/?q=mfold) (Zuker, 2003).
(TIF)

S3 Fig Structure of the representative *AfuSINE2* sequences. Predicted secondary structure was performed using Mfold program (http://mfold.rna.albany.edu/?q=mfold) (Zuker, 2003).
(TIF)

S4 Fig Sequence alignment of individual *AfuSINE3* with *SINE3-1_AO* from *A*. *oryzae*. The alignment which was performed using the Clustal Omega program available at the EMBL-EBI website.
(TIF)

S5 Fig Sequence alignment of 5’ tRNA-related region of individual *AfuSINE2* with tRNA. The alignment which was performed using the Clustal Omega program available at the EMBL-EBI website.
(TIF)

S6 Fig Multiple sequence alignment of the *AfuSINE3* sequences. The 5S rRNA-related and part of the body regions of each sequence were selected for the alignment which was performed using the Clustal Omega program available at the EMBL-EBI website.
(TIF)

S7 Fig Multiple sequence alignment of *AfuSINE3* sequences. The 5S rRNA-unrelated regions of each sequence were selected for the alignment which was performed using the Clustal Omega program available at the EMBL-EBI website.
(TIF)

S8 Fig Multiple sequence alignment of the *AfuSINE3* sequences. The A, IE, and C boxes, which constitute the type 1 pol III promoter, are aligned and highlighted in grey.
(TIF)

S9 Fig Phylogenetic analysis of the *AfuSINE3s*, *A*. *fumigatus* Af293 5S rRNA, *SINE3-1_AO* from *A*. *oryzae* and chicken *DeuSINE3* element (*AmnSINE1_GG*). The 5S rRNA-related regions (nt 1–119) of each sequence were selected for the alignment. A phylogenetic tree was constructed using the fast Fourier transform MAFFT program L9INS-1(2). A bootstrap test was conducted with 1,000 resamplings for the neighbor-joining trees. Numbers on the nodes indicate percentage of bootstrap support from 1,000 replicates with branch lengths indicated.
(TIF)

S10 Fig Multiple sequence alignment of the *AfuSINE2* sequences. The tRNA-related region (nt 1–72) of each sequence was selected for alignment which was performed using the MAFFT with L-INS-i parameter. Potential A and B boxes are highlighted in grey.
(TIF)

S11 Fig Phylogenetic analysis of the *AfuSINE2* sequences and other tRNA-derived *SINEs*. The tRNA-related region (nt 1–72) of each sequence was selected for the alignment. A phylogenetic tree was constructed using the fast Fourier transform MAFFT program L9INS-1(2). A bootstrap test was conducted with 1,000 resamplings for the neighbor-joining trees. Numbers on the nodes indicate percentage of bootstrap support from 1,000 replicates with branch lengths indicated. *MIRc* is a *SINE2/tRNA* (Pro) from mammals, *Foxy* is a tRNA-derived *SINE* from *Fusarium oxysporum* f.sp. *lycopersici*, *SINE2-1_BG* is a *SINE2/tRNA* (Gly) from barley powdery mildew *Blumeria graminis*, *MgSINE* is a tRNA-derive *SINE* from *Magnaporthe grisea*, and *LFSINE_Vert* is a *SINE2/tRNA* (His) from Latimeria.
(TIF)

S12 Fig Agarose gel electrophoresis of the PCR and RT-PCR products of the 13 candidate *AfuSINE*. Agarose gel electrophoresis showing the PCR and RT-PCR products of the 13 candidate *AfuSINE* sequences; Lane 1 for each *AfuSINE* shows PCR amplicons generated from genomic DNA; Lane 2 shows for each *AfuSINE*, amplicons generated following RT-PCR; Lane 3 shows for each *AfuSINE*, amplicons generated from (-RT) negative controls RT-PCR. Hyperladder 1 (M; 10 kbp; Bioline) and Quick-Load® 100 bp DNA Ladder (NEB) were used as markers. Electrophoretic analysis was performed in 2.5% agarose gels for 3 h at 80 V.
(TIF)

S1 Table Oligonucleotide primers used for PCR and RT-PCR amplification of *A*. *fumigatus* Af293 *SINE* sequences. (PDF)

## References

[1] Bradshaw VA and McEntee K. DNA damage activates transcription and transposition of yeast *Ty* retrotransposons. Mol Gen Genet. 1989; 218: 465–474. 2555668 10.1007/BF00332411

[2] Paquin CE and Williams VM. In: Eukaryotic transposable elements as mutagenic agents, Banbury Report 30, Cold Spring Harbor Laboratory, Cold Spring Harbor, NY; 1988.

[3] Wessler SR. Turned on by stress. Plant retrotransposons. Curr Biol. 1996; 6: 959–961. 8805314 10.1016/s0960-9822(02)00638-3

[4] Grandbastien MA. Activation of plant retrotransposons under stress conditions. Trends Plant Sci. 1998; 3: 181–187.

[5] Capy P, Gasperi G, Biémont C and Bazin C. Stress and transposable elements: Co-evolution or useful parasites? Heredity. 2000; 85: 101–106. 11012710 10.1046/j.1365-2540.2000.00751.x

[6] Kramerov DA and Vassetzky NS. SINEs. WIRs:RNA. 2011; 2(6): 772–786. 10.1002/wrna.91 21976282

[7] Mes JJ, Haring MA and Cornelissen BJ. *Foxy*: an active family of short interspersed nuclear elements from *Fusarium oxysporum*. Mol Gen Genet. 2000; 263: 271–280. 10778745 10.1007/pl00008681

[8] Kapitonov VV and Jurka J. A novel class of *SINE* elements derived from 5S RNA. Mol Biol Evol. 2003; 20: 694–702. 10.1093/molbev/msg075 12679554

[9] Kramerov DA and Vassetzky NS. Origin and evolution of SINEs in eukaryotic genomes. Heredity. 2011; 107: 487–495. 10.1038/hdy.2011.43 21673742 PMC3242629

[10] Kachroo P, Leong SA and Chatto BB. *Mg-SINE*: A short interspersed nuclear element from the rice blast fungus, *Magnaporthe grisea*. Proc Natl Acad Sci USA. 1995; 92: 11125–11129. 7479950 10.1073/pnas.92.24.11125 PMC40584

[11] Sone T, Suto M and Tomita F. Host species-specific repetitive DNA sequence in the genome of *Magnaporthe grisea*, the rice blast fungus. Biosci Biotechnol Biochem. 1993; 57: 1228–1230. 10.1271/bbb.57.1228 7765312

[12] Wei YD, Collinge DB, Smeregaard-Peterson V and Thordal-Christensen H. Characterization of the transcript of a new class of retroposon-type repetitive element cloned from the powdery mildew fungus *Erysiphe graminis*. Mol Gen Genet. 1996; 250: 477–482. 8602165 10.1007/BF02174036

[13] Rasmussen M, Rossen L and Giese H. *SINE*-like properties of a highly repetitive element in the genome of the obligateparasitic fungus *Erysiphe graminis* f. sp. *hordei*. Mol Gen Genet. 1993; 239: 298–303. 8510659 10.1007/BF00281631

[14] Kim HG, Meinhardt LW, Benny U and Kistler HC. *Nrs*I, a repetitive element linked to pisatin demethylase genes on a dispensable chromosome of *Nectria haematococca*. Mol Plant Microbe Interactions. 1995; 8: 524–531. 10.1094/mpmi-8-0524 8589408

[15] Spanu PD, Abbott JC, Amsalem J, Burgis TA, Soanes DM, Stuber K, et al Genome expansion and gene loss in powdery mildew fungi reveal trade-offs in extreme parasitism. Science. 2010; 330: 1543–1546. 10.1126/science.1194573 21148392

[16] Bao W and Jurka J. *SINEs* from barley powdery mildew. Repbase Reports. 2011; 11(9): 2582–2582.

[17] Whisson SC, Avrova AO, Lavrova O and Pritchard L. Families of short interspersed elements in the genome of the oomycete plant pathogen, *Phytophthora infestans*. Fungal Genet Biol. 2005; 42: 351–365. 10.1016/j.fgb.2005.01.004 15749054

[18] O’Gorman CM, Fuller HT and Dyer PS. Discovery of a sexual cycle in the opportunistic fungal pathogen *Aspergillus fumigatus*. Nature. 2009; 457: 471–474. 10.1038/nature07528 19043401

[19] Latgé JP. *Aspergillus fumigatus* and Aspergillosis. Clin Microbio. Rev. 1999; 12(2): 310–350. 10.1128/cmr.12.2.310 PMC88920 10194462

[20] Galagan JE, Calvo SE, Cuomo C, Ma LJ, Wortman JR, Batzoglou S, et al Sequencing of *Aspergillus nidulans* and comparative analysis with *A*. *fumigatus* and *A*. *oryzae*. Nature. 2005; 438: 1105–1115. 10.1038/nature04341 16372000

[21] Kapitonov VV and Jurka J. *SINE3-1_AO*, a family of 5S rRNA-derived nonautonomous non-LTR retrotransposons in the *Aspergillus oryzae* genome. Repbase Reports. 2006; 6: 45–45.

[22] Huber F and Bignell E. Distribution, expression and expansion of *Aspergillus fumigatus LINE*-like retrotransposon populations in clinical and environmental isolates. Fungal Genet Biol. 2014; 64: 36–44. 10.1016/j.fgb.2014.01.002 24440682

[23] Mabey JE, Anderson MJ, Giles PF, Miller CJ, Attwood TK, Paton NW, et al CADRE: the central *Aspergillus* data Repository 2012. Nucleic Acids Res. 2012; 40: D660–6. 10.1093/nar/gkr971 22080563 PMC3245145

[24] Arnaud MB, Chibucos MC, Costanzo MC, Crabtree J, Inglis DO, Lotia A, et al The *Aspergillus* Genome Database, a curated comparative genomics resource for gene, protein and sequence information for the *Aspergillus* research community. Nucleic Acids Res. 2010; 38: D420–7. 10.1093/nar/gkp751 19773420 PMC2808984

[25] Kohany O, Gentles AJ, Hankus L and Jurka J. Annotation, submission and screening of repetitive elements in Repbase: Repbase Submittor and Censor. BMC Bioinformatics. 2006; 7: 474 10.1186/1471-2105-7-474 17064419 PMC1634758

[26] Smit AFA, Hubley R and Green P. RepeatMasker Open-4.0. 2013–2015. Available: www.repeatmasker.org

[27] Jurka J. Repbase update: a database and an electronic journal of repetitive elements. Trends in Genetics. 2000; 16: 418–420. 10973072 10.1016/s0168-9525(00)02093-x

[28] Lawe TM and Eddy SR. tRNAscan-SE: A program for improved detection of transfer RNA genes in genomic sequence. Nucleic Acids Res. 1997; 25: 955–964. 9023104 10.1093/nar/25.5.955 PMC146525

[29] McWilliam H, Li W, Uludag M, Squizzato S, Park YM, Buso N, et al Analysis tool web services from the EMBL-EBI. Nucleic Acids Res. 2013; 41 (Web Server issue):W597–600. 10.1093/nar/gkt376 23671338 PMC3692137

[30] Katoh K and Standley DM. MAFFT multiple sequence alignment software version 7: Improvements in performance and usability. Mol Biol Evol. 2013; 30(4): 772–780. 10.1093/molbev/mst010 23329690 PMC3603318

[31] Kanhayuwa L, Kotta-Luizou I, Ozkan S, Gunning AP and Coutts RHA. A novel mycovirus from *Aspergillus fumigatus* contains four unique dsRNAs as its genome and is infectious as dsRNA. Proc Natl Acad Sci USA. 2015; 112: 9100–9105. 10.1073/pnas.1419225112 26139522 PMC4517262

[32] Pontecorvo G, Roper JA, Hemmons LM, Macdonald KD and Bufton AW. The genetics of *Aspergillus nidulans*. Adv Genet. 1953; 5: 141–238. 13040135 10.1016/s0065-2660(08)60408-3

[33] Lu C, Meyers BC and Green PJ. Construction of small RNA cDNA libraries for deep sequencing. Methods. 2007; 43: 110–117. 10.1016/j.ymeth.2007.05.002 17889797

[34] Pall GS, Codony-Servat C, Byrne J, Ritchie L and Hamilton A. Carbodiimide-mediated cross-linking of RNA to nylon membrane improves the detection of siRNA, miRNA and piRNA by northern blot. Nucleic Acids Res. 2007; 35: e60, 10.1093/nar/gkm112 17405769 PMC1885651

[35] Kreuze JF, Savenkov EI, Cuellar W, Li X and Valkonen JPT. Viral class 1 RNase III involved in suppression of RNA silencing. J Virol. 2005; 79: 7227–7238. 10.1128/JVI.79.11.7227-7238.2005 15890961 PMC1112141

[36] Zuker M. Mfold web server for nucleic acid folding and hybridization prediction. Nucleic Acids Res. 2003; 31: 3406–3415. 12824337 10.1093/nar/gkg595 PMC169194

[37] Borodulina OR and Kramerov D A. Wide distribution of short interspersed elements among eukaryotic genomes. FEBS Letters. 1999; 457: 409–413. 10471819 10.1016/s0014-5793(99)01059-5

[38] Fantaccione S, Woodrow P and Pontecorvo G. Identification of a family of *SINEs* and *LINEs* in the *Pipistrellus kuhli* genome: A new structure and functional symbiotic relationship. Genomics. 2008; 91: 178–185. 10.1016/j.ygeno.2007.10.008 18068947

[39] Koval AP, Veniaminova NA and Kramerov DA. Additional B box of RNA polymerase III promoter in SINE B1 can be functional. Gene. 2011; 487(2): 113–117. 10.1016/j.gene.2011.08.001 21855615

[40] Smit AF and Riggs AD. *MIRs* are classic, tRNA‐derived *SINEs* that amplified before the mammalian radiation. Nucleic Acids Res. 1995; 23: 98–102. 7870595 10.1093/nar/23.1.98 PMC306635

[41] Neuveglise C, Sarfati J, Latge JP and Paris S. *Afut1*, a retrotransposon-like element from *Aspergillus fumigatus*. Nucleic Acids Res. 1996; 24: 1428–1434. 8628674 10.1093/nar/24.8.1428 PMC145799

[42] Ogiwara I, Miya M, Ohshima K and Okada N. Retropositional parasitism of SINEs on LINEs: Identification of SINEs and LINEs in Elasmobranchs.Mol Biol Evol. 1999; 16(9): 1238–1250. 10486979 10.1093/oxfordjournals.molbev.a026214

[43] Hammond TM, Andrewski MD, Roossinck MJ and Keller NP. *Aspergillus* mycoviruses are targets and suppressors of RNA silencing. Eukaryot Cell. 2008; 7(2): 350–357. 10.1128/EC.00356-07 18065651 PMC2238147

[44] Brookfield JF and Badge RM. Population genetics models of transposable elements. Genetica. 1997; 100: 281–294. 9440281

[45] Daniels GR and Deininger PL. A second major class of *Alu* family repeated DNA sequences in a primate genome. Nucleic Acids Res. 1983; 11: 7595–7610. 6647032 10.1093/nar/11.21.7595 PMC326505

[46] Fantaccione S, Russo C, Palomba P, Rienzo M and Pontecorvo G. A new pair of *CR1*-like *LINE* and tRNA-derived *SINE* in *Podarcis sicula* genome. Gene. 2004; 339: 189–198. 10.1016/j.gene.2004.06.051 15363859

