# Supplementary figures and images for: Short Interspersed Nuclear Element (SINE) Sequences in the Genome of the Human Pathogenic Fungus Aspergillus fumigatus Af293
Source: PLoS One. 2016 Oct 13;11(10):e0163215. doi: 10.1371/journal.pone.0163215 (PMC5063351; doi:10.1371/journal.pone.0163215)

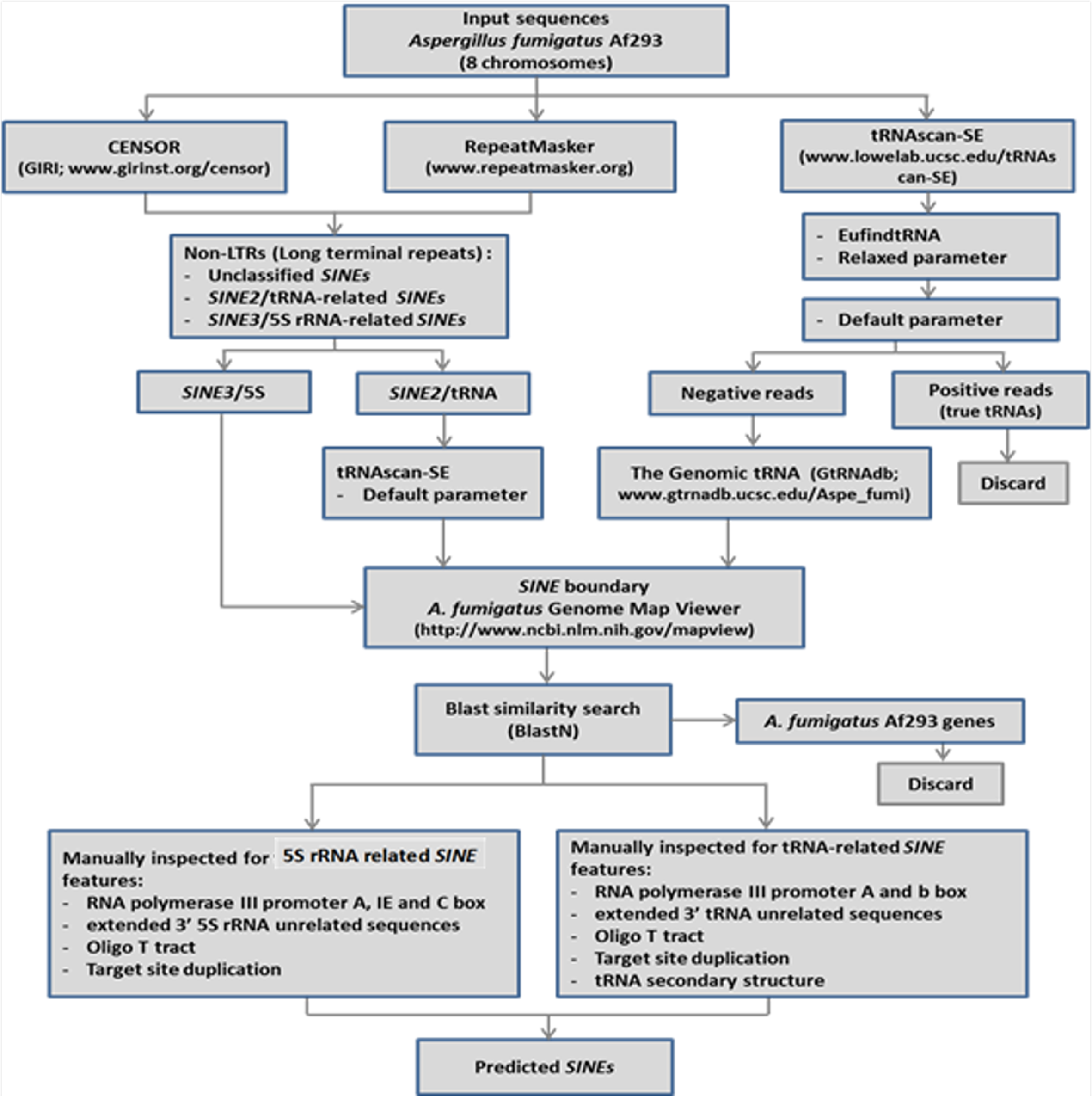

Supplement: S1 Fig — (TIF) [file pone.0163215.s001.tif]

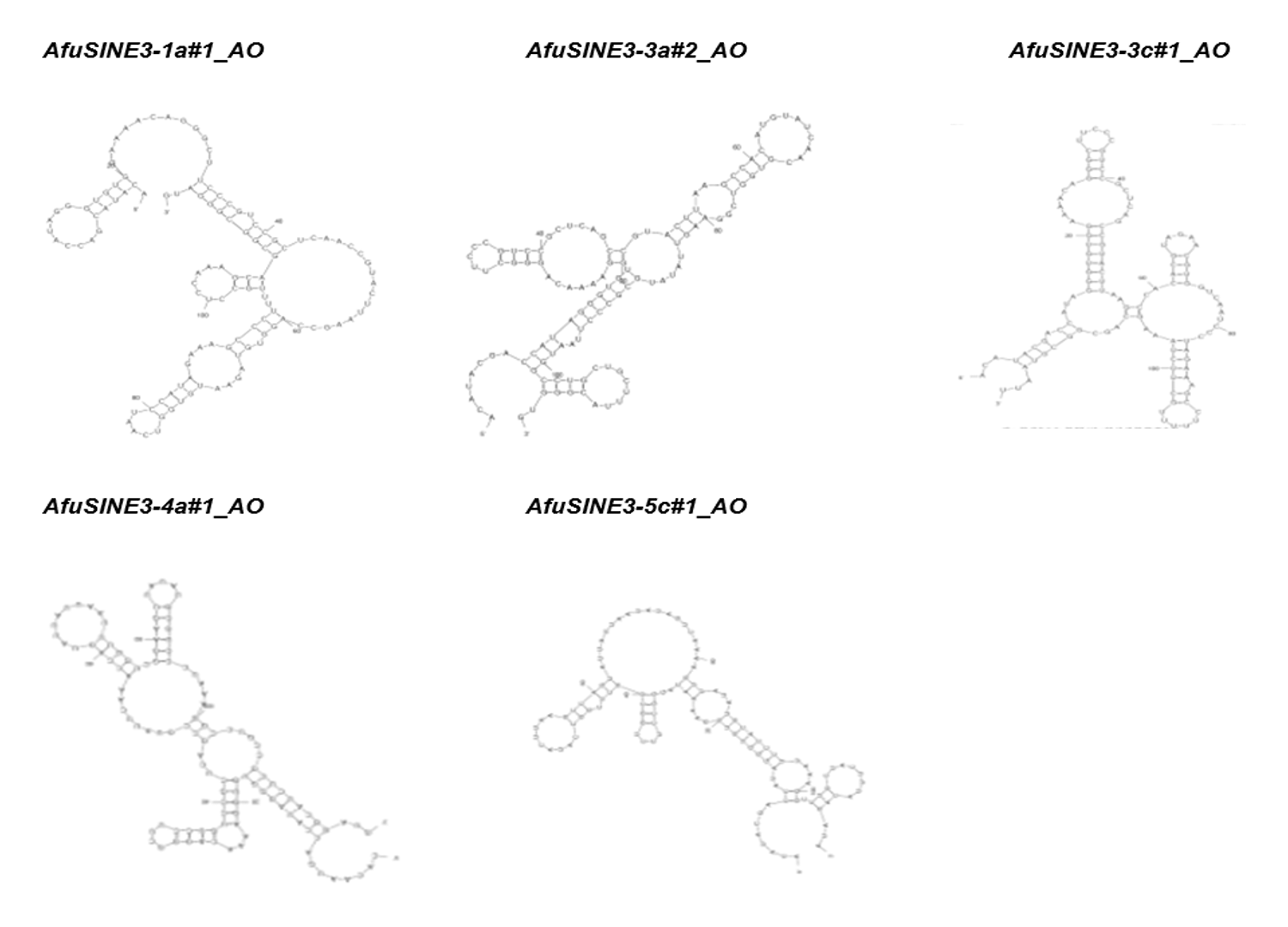

Supplement: S2 Fig — Predicted secondary structure was performed using Mfold program (http://mfold.rna.albany.edu/?q=mfold) (Zuker, 2003). (TIF) [file pone.0163215.s002.tif]

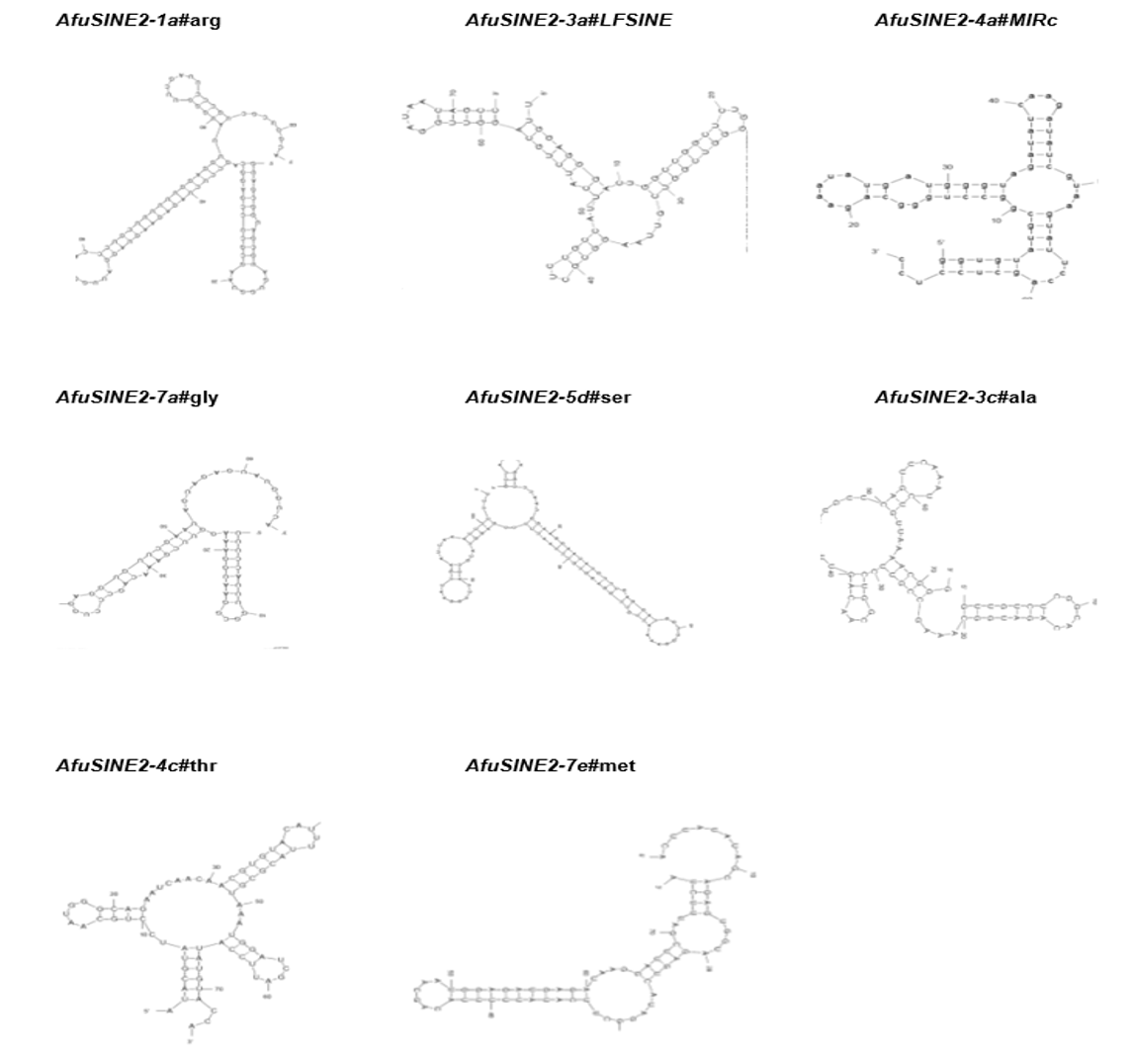

Supplement: S3 Fig — Predicted secondary structure was performed using Mfold program (http://mfold.rna.albany.edu/?q=mfold) (Zuker, 2003). (TIF) [file pone.0163215.s003.tif]

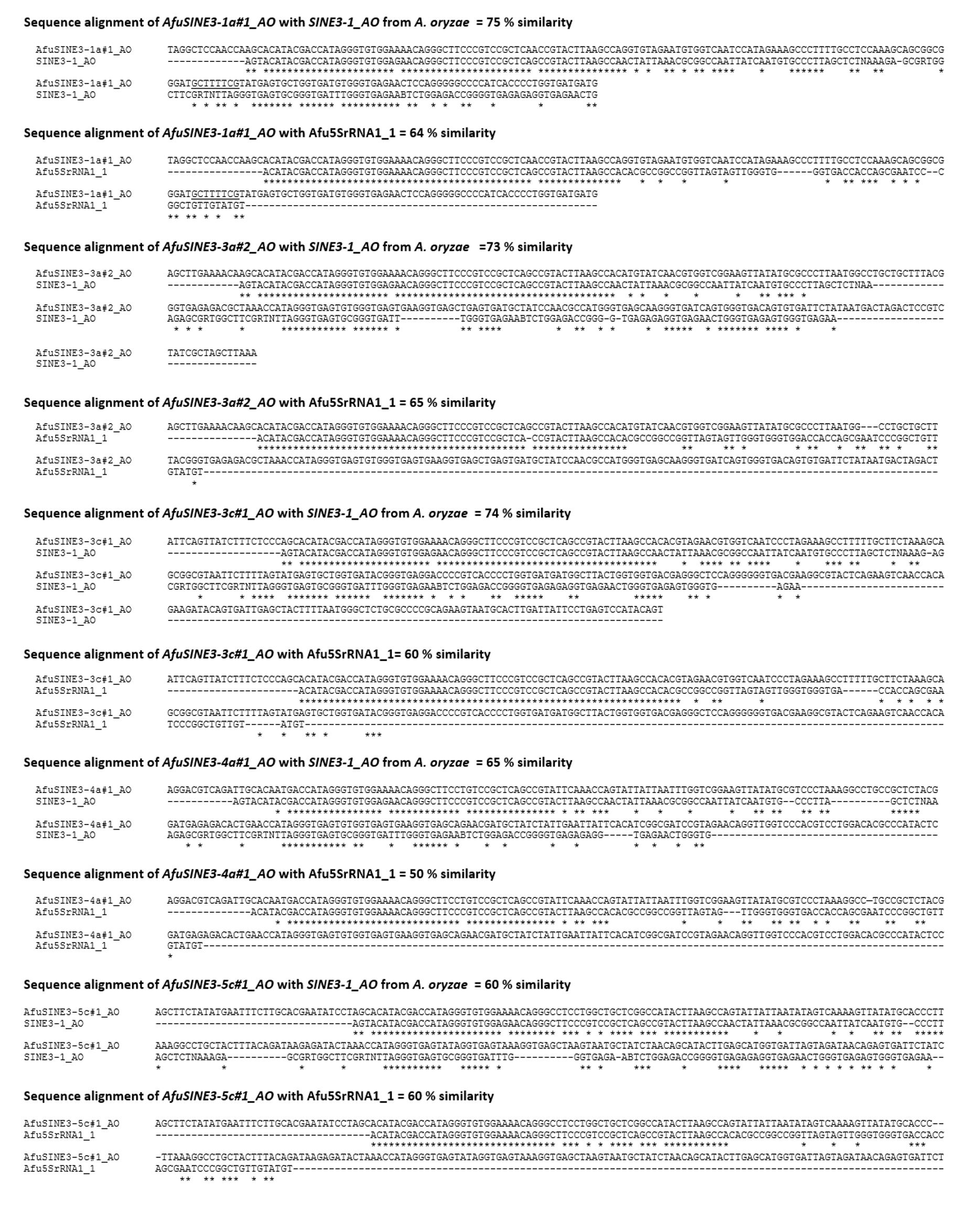

Supplement: S4 Fig — The alignment which was performed using the Clustal Omega program available at the EMBL-EBI website. (TIF) [file pone.0163215.s004.tif]

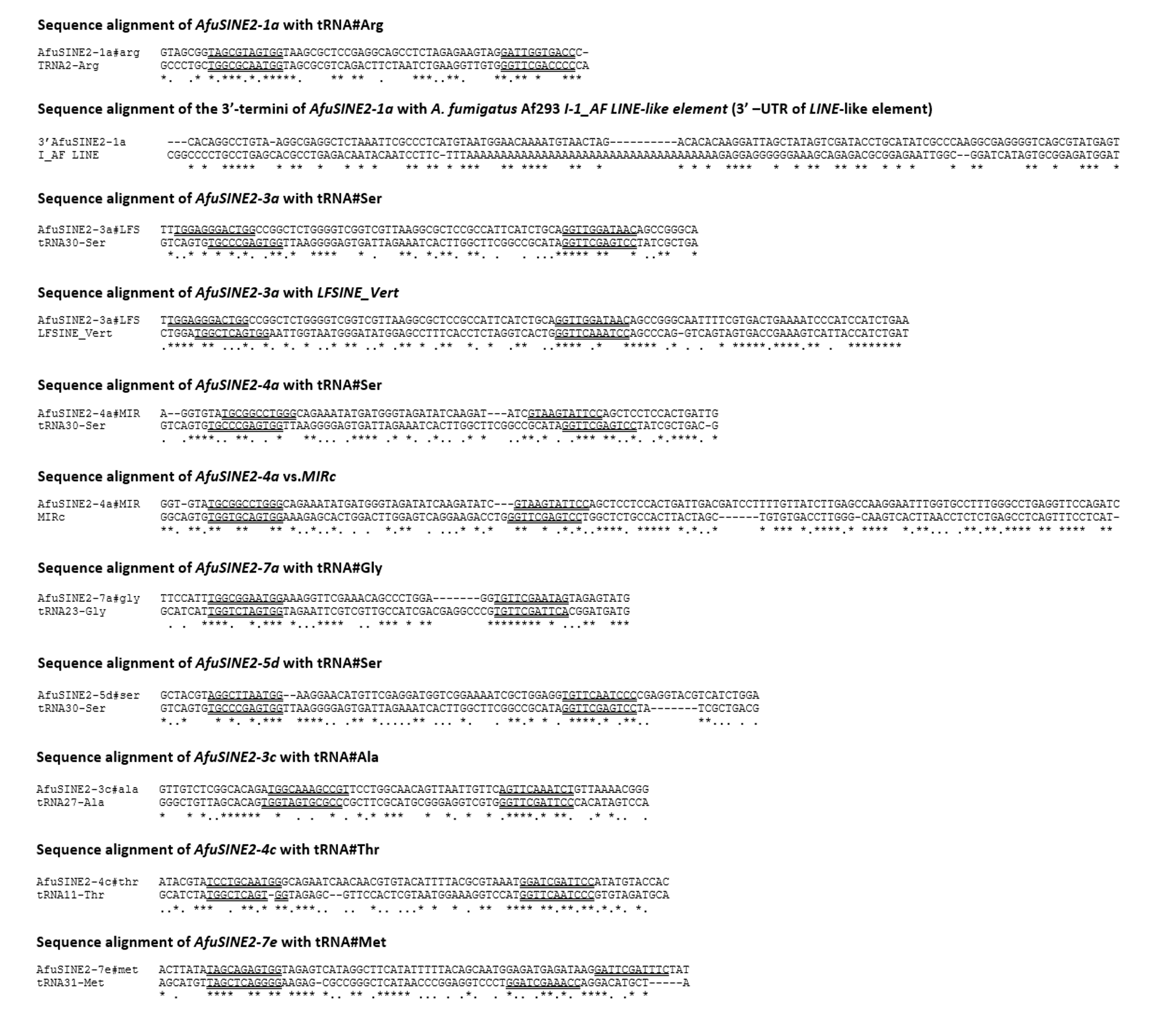

Supplement: S5 Fig — The alignment which was performed using the Clustal Omega program available at the EMBL-EBI website. (TIF) [file pone.0163215.s005.tif]

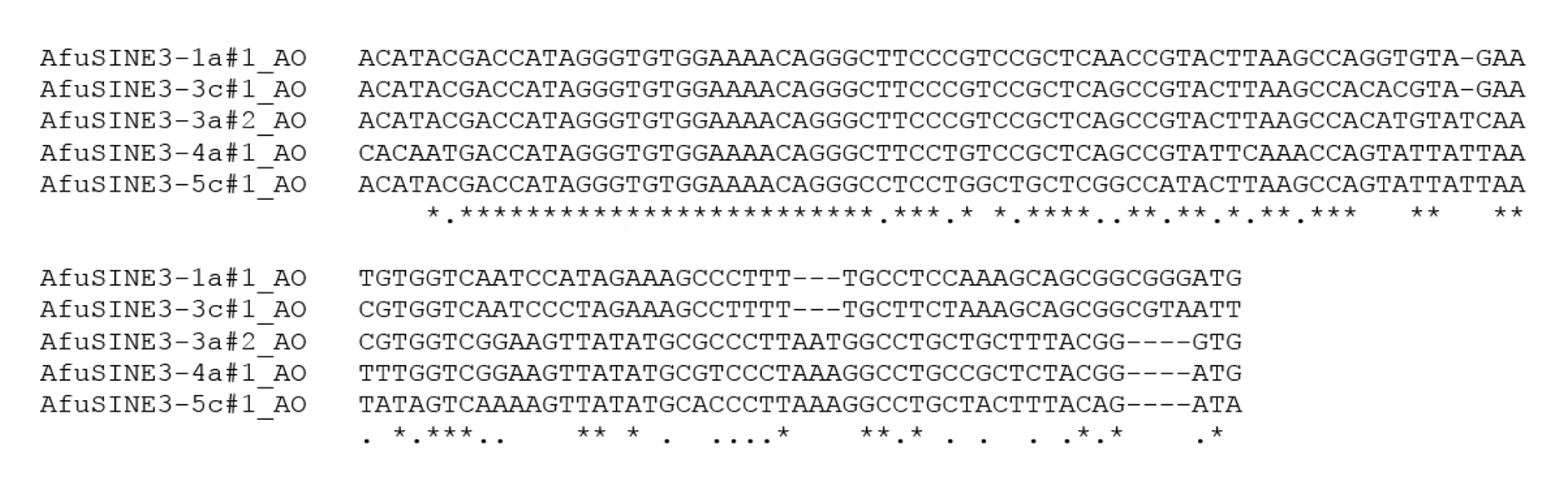

Supplement: S6 Fig — The 5S rRNA-related and part of the body regions of each sequence were selected for the alignment which was performed using the Clustal Omega program available at the EMBL-EBI website. (TIF) [file pone.0163215.s006.tif]

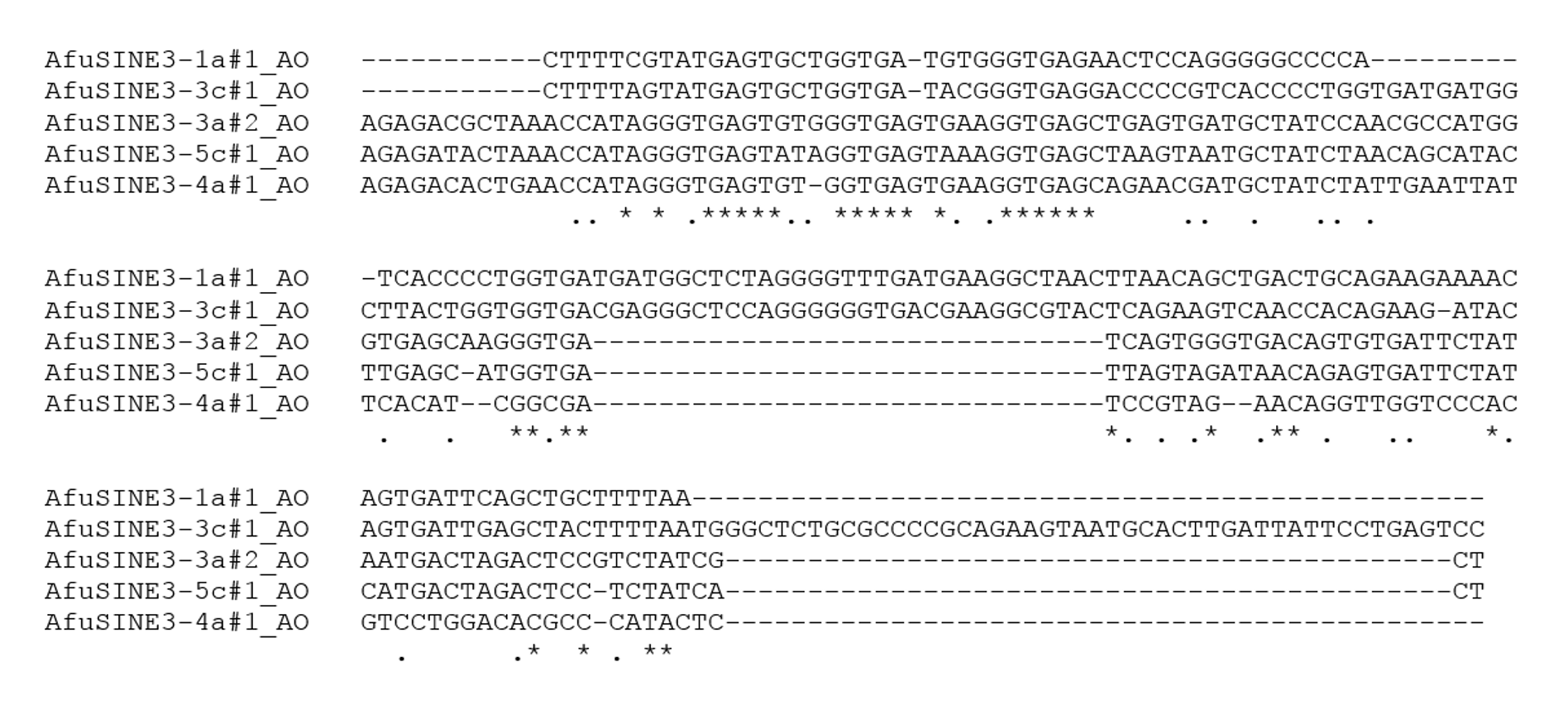

Supplement: S7 Fig — The 5S rRNA-unrelated regions of each sequence were selected for the alignment which was performed using the Clustal Omega program available at the EMBL-EBI website. (TIF) [file pone.0163215.s007.tif]

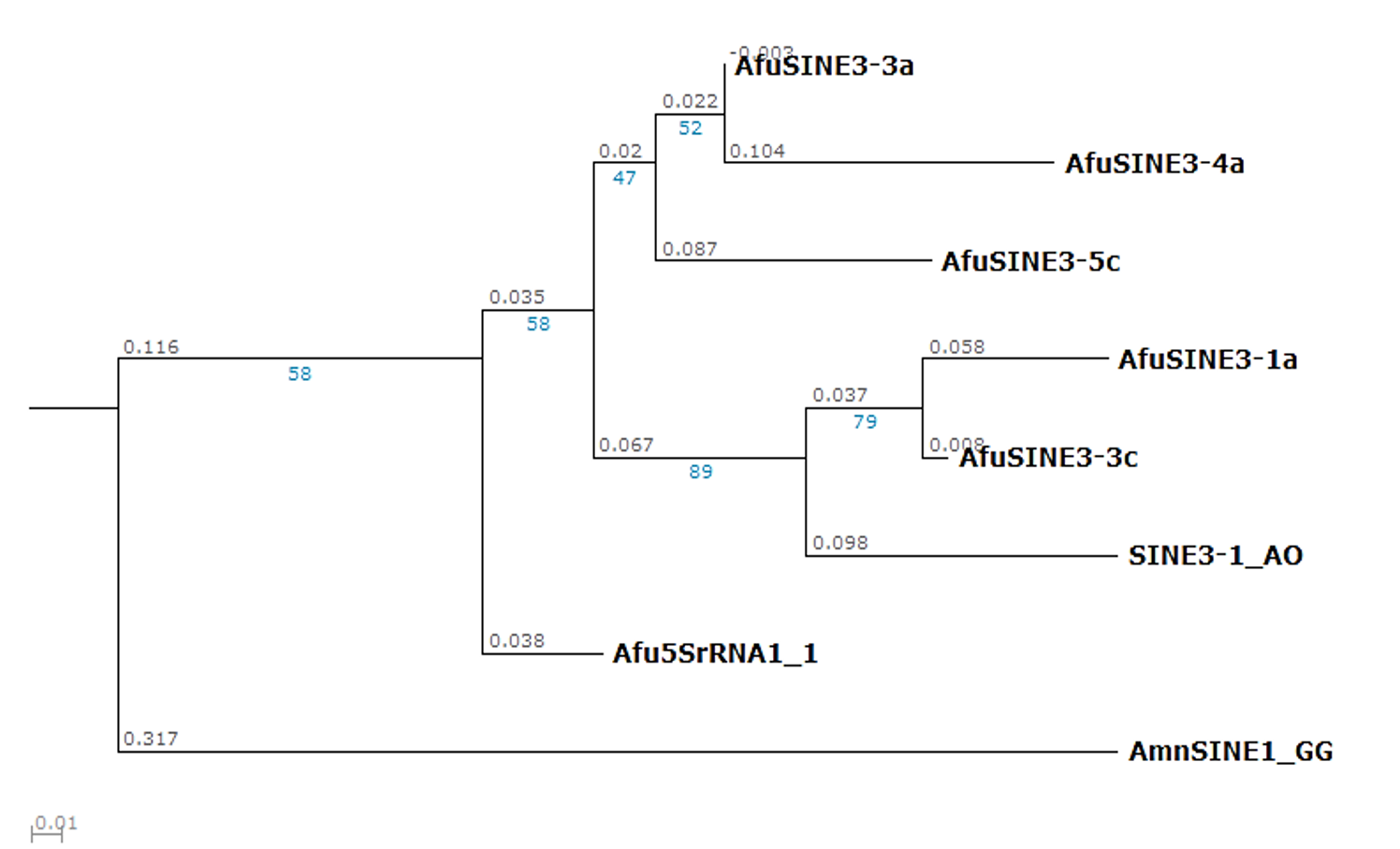

Supplement: S9 Fig — The 5S rRNA-related regions (nt 1–119) of each sequence were selected for the alignment. A phylogenetic tree was constructed using the fast Fourier transform MAFFT program L9INS-1(2). A bootstrap test was conducted with 1,000 resamplings for the neighbor-joining trees. Numbers on the nodes indicate percentage of bootstrap support from 1,000 replicates with branch lengths indicated. (TIF) [file pone.0163215.s009.tif]

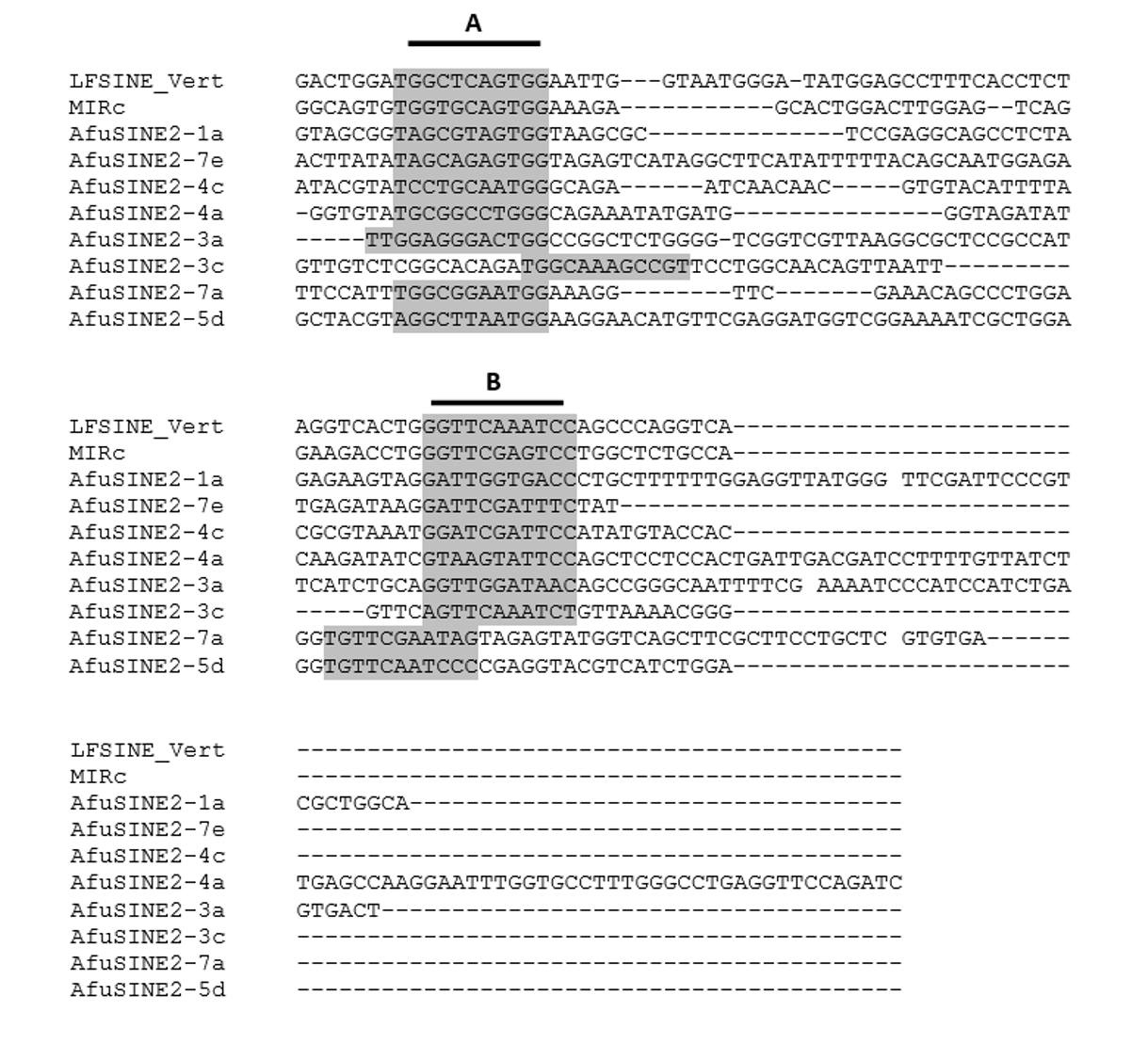

Supplement: S10 Fig — The tRNA-related region (nt 1–72) of each sequence was selected for alignment which was performed using the MAFFT with L-INS-i parameter. Potential A and B boxes are highlighted in grey. (TIF) [file pone.0163215.s010.tif]

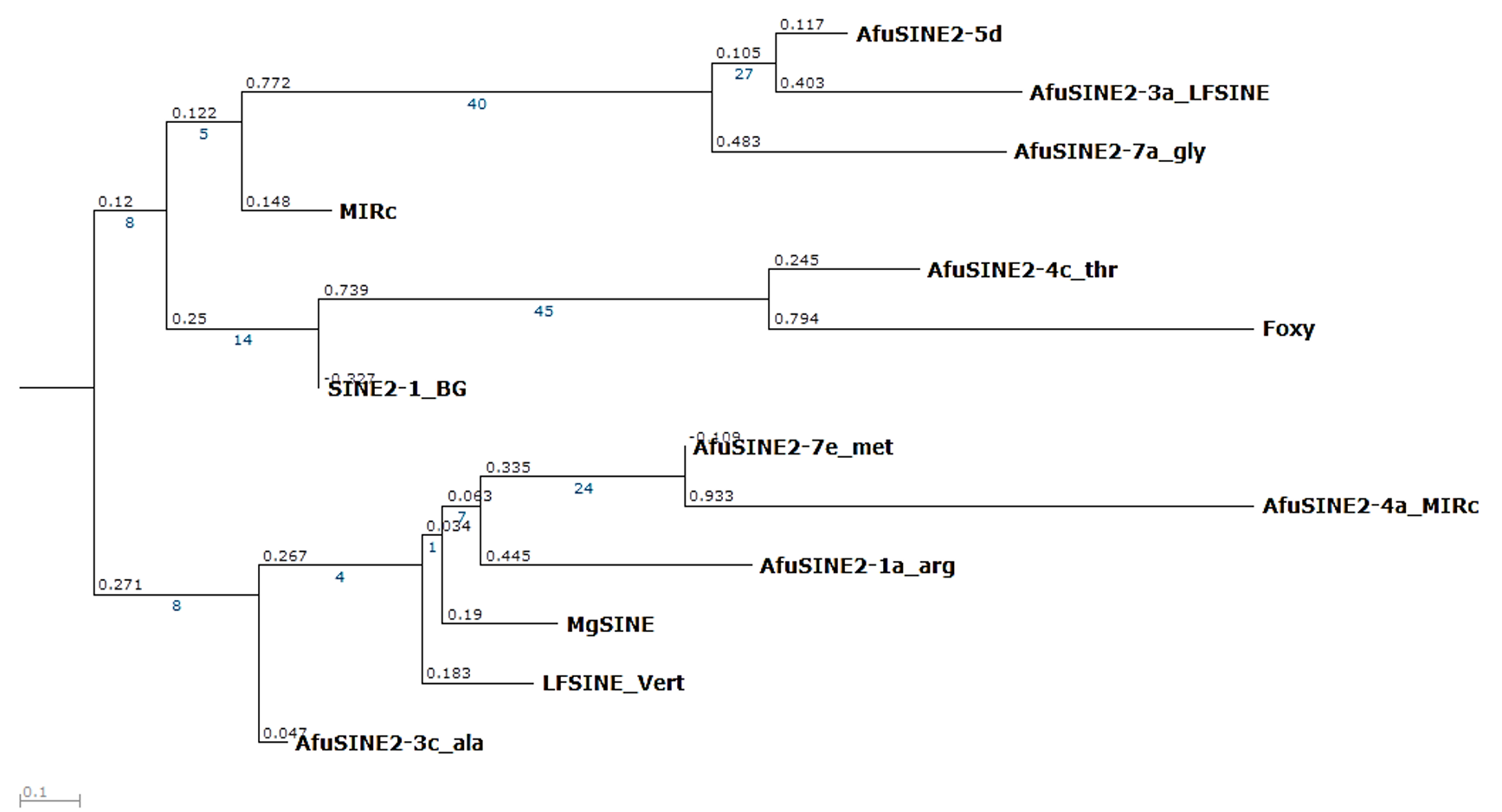

Supplement: S11 Fig — The tRNA-related region (nt 1–72) of each sequence was selected for the alignment. A phylogenetic tree was constructed using the fast Fourier transform MAFFT program L9INS-1(2). A bootstrap test was conducted with 1,000 resamplings for the neighbor-joining trees. Numbers on the nodes indicate percentage of bootstrap support from 1,000 replicates with branch lengths indicated. MIRc is a SINE2/tRNA (Pro) from mammals, Foxy is a tRNA-derived SINE from Fusarium oxysporum f.sp. lycopersici, SINE2-1_BG is a SINE2/tRNA (Gly) from barley powdery mildew Blumeria graminis, MgSINE is a tRNA-derive SINE from Magnaporthe grisea, and LFSINE_Vert is a SINE2/tRNA (His) from Latimeria. (TIF) [file pone.0163215.s011.tif]

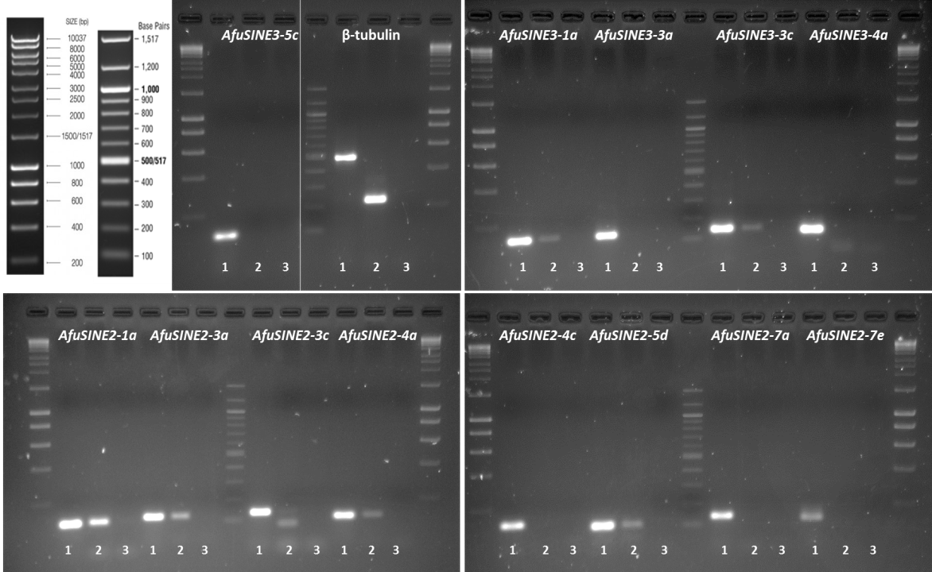

Supplement: S12 Fig — Agarose gel electrophoresis showing the PCR and RT-PCR products of the 13 candidate AfuSINE sequences; Lane 1 for each AfuSINE shows PCR amplicons generated from genomic DNA; Lane 2 shows for each AfuSINE, amplicons generated following RT-PCR; Lane 3 shows for each AfuSINE, amplicons generated from (-RT) negative controls RT-PCR. Hyperladder 1 (M; 10 kbp; Bioline) and Quick-Load® 100 bp DNA Ladder (NEB) were used as markers. Electrophoretic analysis was performed in 2.5% agarose gels for 3 h at 80 V. (TIF) [file pone.0163215.s012.tif]
