# Supplementary material for: Short Interspersed Nuclear Element (SINE) Sequences in the Genome of the Human Pathogenic Fungus Aspergillus fumigatus Af293
Source: PLoS One. 2016 Oct 13;11(10):e0163215. doi: 10.1371/journal.pone.0163215 (PMC5063351; doi:10.1371/journal.pone.0163215)
Supplement: S1 Table — (PDF) [file pone.0163215.s013.pdf]

AfuSINEs	Primers	Primer sequences	Product size (bp)
AfuSINE3-1a	SINE3F	5’-GACCATAGGGTGTGGAAAAC -3’	132
	SINE3R1a	5’-CCAGCACTCATACGAAAAGCA-3’	
AfuSINE3-3a	SINE3F	5’-GACCATAGGGTGTGGAAAAC-3’	133
	SINE3R3a	5’-CCCTATGGTTTAGCGTCTCTCA-3’	
AfuSINE3-3c	SINE3F	5’-GACCATAGGGTGTGGAAAAC-3’	144
	SINE3R3c	5’-CCCGTATCACCAGCACTCAT-3’	
AfuSINE3-4a	SINE3F	5’-GACCATAGGGTGTGGAAAAC-3’	133
	SINE3R4a	5’-CCCTATGGTTCAGTGTCTCTCA-3’	
AfuSINE3-5c	SINE3F	5’-GACCATAGGGTGTGGAAAAC-3’	112
	SINE3R5c	5’-ATCTGTAAAGTAGCAGGCCTTT-3’	
AfuSINE2-1a	SINE2F1a	5’-TAGTGGTAAGCGCTCCGAG-3’	102
	SINE2R1a	5’-GGTAAATGTTGTGCCAGCGA-3’	
AfuSINE2-3a	SINE2F3a	5’-CTCTGGGGTCGGTCGTTAAG-3’	120
	SINE2R3a	5’-GTCGGCTTTCAACATACTCACA-3’	
AfuSINE2-4a	SINE2F4a	5’-CCTGGGCAGAAATATGATGGG-3’	110
	SINE2R4a	5’-CCCAAAGGCACCAAATTCCT-3’	
AfuSINE2-7a	SINE2F7a	5’-GCGGAATGGAAAGGTTCGAA-3’	139
	SINE2R7a	5’-GAATAGAATGCGGCCCACTG-3’	
AfuSINE2-5d	SINE2F5d	5’-GAGGATGGTCGGAAAATCGC-3’	101
	SINE2R5d	5’-ACCTAATACTCTCAGCTCTCCC-3’	
AfuSINE2-3c	SINE2F3c	5’-GTTGTCTCGGCACAGATGG-3’	134
	SINE2R3c	5’-TCACTGGGTCTTTTGGCTAGA-3’	
AfuSINE2-4c	SINE2F4c	5’-GGGCAGAATCAACAACGTGT-3’	123
	SINE2R4c	5’-TTGGTAAGTGTTTCGGTGGC-3’	
AfuSINE2-7e	SINE2F7e	5’-ACTTATATAGCAGAGTGGTA-3’	123
	SINE2R7e	5’-TCCTATAGCTTCCCTTAAAG-3’	
β-tubulin	TUB-For	5’-CGACGCGAGACTGTGTTAAG-3’	440 (PCR)
	TUB-Rev	5’-TAGTTGGTGCTCAAGGATGG-3’	210 (RT-PCR)
